# Reactive astrocytes acquire neuroprotective as well as deleterious signatures in response to Tau and Aß pathology

**DOI:** 10.1038/s41467-021-27702-w

**Published:** 2022-01-10

**Authors:** Zoeb Jiwaji, Sachin S. Tiwari, Rolando X. Avilés-Reyes, Monique Hooley, David Hampton, Megan Torvell, Delinda A. Johnson, Jamie McQueen, Paul Baxter, Kayalvizhi Sabari-Sankar, Jing Qiu, Xin He, Jill Fowler, James Febery, Jenna Gregory, Jamie Rose, Jane Tulloch, Jamie Loan, David Story, Karina McDade, Amy M. Smith, Peta Greer, Matthew Ball, Peter C. Kind, Paul M. Matthews, Colin Smith, Owen Dando, Tara L. Spires-Jones, Jeffrey A. Johnson, Siddharthan Chandran, Giles E. Hardingham

**Affiliations:** 1 UK Dementia Research Institute at the University of Edinburgh, Chancellor’s Building, Edinburgh Medical School, Edinburgh, EH16 4SB UK; 2 Centre for Discovery Brain Sciences, University of Edinburgh, Hugh Robson Building, George Square, Edinburgh, EH8 9XD UK; 3 Centre for Clinical Brain Sciences, University of Edinburgh Chancellor’s Building, Edinburgh, UK; 4 Division of Pharmaceutical Sciences, School of Pharmacy, University of Wisconsin-Madison, Madison, WI USA; 5 UK Dementia Research Institute at Cardiff University, Hadyn Ellis Building, Cardiff, CF24 4HQ UK; 6 UK Dementia Research Institute at Imperial College, Burlington Danes Building, Hammersmith Hospital, London, W12 0NN UK; 7 Simons Initiative for the Developing Brain, University of Edinburgh, Hugh Robson Building, George Square, Edinburgh, EH8 9XD UK

**Keywords:** Transcriptomics, Alzheimer's disease, Astrocyte, Molecular neuroscience

## Abstract

Alzheimer’s disease (AD) alters astrocytes, but the effect of Aß and Tau pathology is poorly understood. TRAP-seq translatome analysis of astrocytes in APP/PS1 ß-amyloidopathy and MAPT^P301S^ tauopathy mice revealed that only Aß influenced expression of AD risk genes, but both pathologies precociously induced age-dependent changes, and had distinct but overlapping signatures found in human post-mortem AD astrocytes. Both Aß and Tau pathology induced an astrocyte signature involving repression of bioenergetic and translation machinery, and induction of inflammation pathways plus protein degradation/proteostasis genes, the latter enriched in targets of inflammatory mediator Spi1 and stress-activated cytoprotective Nrf2. Astrocyte-specific Nrf2 expression induced a reactive phenotype which recapitulated elements of this proteostasis signature, reduced Aß deposition and phospho-tau accumulation in their respective models, and rescued brain-wide transcriptional deregulation, cellular pathology, neurodegeneration and behavioural/cognitive deficits. Thus, Aß and Tau induce overlapping astrocyte profiles associated with both deleterious and adaptive-protective signals, the latter of which can slow patho-progression.

## Abstract (web summary)

Alzheimer’s disease is associated with changes in astrocytes. Here the authors investigated the astrocyte translatome associated with amyloid-ß and tau pathology.

## Introduction

Alzheimer’s disease (AD) causes progressive cognitive, memory and language impairment, along with neurodegeneration and brain atrophy. It is histopathologically defined by the presence of amyloid-beta-(Aß) containing plaques and tau-containing neurofibrillary tangles, with other consistent hallmarks being dystrophic neurites, neuronal loss and reactive astrogliosis^1,2^. Astrocytes play a fundamental role in the healthy brain, playing myriad roles including synaptogenesis, neurovascular coupling, neuronal bioenergetic support, protein and waste clearance, maintenance of redox, pH, and K+ balance as well as neurotransmitter homoeostasis^3^. Alois Alzheimer himself noted changes to astrocytes in AD. However, the molecular changes that take place in astrocytes during AD, and the consequences for disease trajectory, remain incompletely understood^4^. Interrogation of post-mortem brain tissue has revealed some changes to astrocytes in AD^5–7^ but this approach primarily delivers insight into advanced AD, reflecting the consequence of decades of patho-progression involving neurodegeneration, neuroinflammation and metabolic deficits, rather than early changes. This is of particular relevance since imaging studies suggest that astrogliosis may be a relatively early event in the disease^8^. Moreover, despite the catch-all term of ‘astrogliosis’ it is becoming clear that the properties of ‘reactive astrocytes’ vary substantially according to the upstream trigger and evolve substantially during patho-progression, reflecting the diversity of extracellular signals being received^9^.While some signals can induce deleterious effects, others may trigger adaptive-protective consequences, and so a reactive astrocyte phenotype cannot be predicted from simple marker gene expression (e.g. Gfap). Another unanswered question is how the two aspects of AD-associated proteopathy, Aß and Tau, influence astrocytes. Aß pathology is thought to occur first in prodromal AD, and lead to tau pathology, a pre-requisite for synapse loss and neurodegeneration. Mouse models of ß-amyloidopathy and tauopathy provide the opportunity to study the effects of these two types of pathology on astrocytes and to identify the common effects, as well as any differences. We set out to test the hypothesis that Aß and Tau trigger distinct but overlapping responses in astrocytes, identifying the induction of both putative deleterious and adaptive-protective signatures, the latter of which is able to attenuate these AD-relevant pathologies if pre-emptively activated.

## Results

### Astrocyte TRAP-seq in mouse models of tauopathy and ß-amyloidopathy

The MAPT^P301S^ tauopathy mouse model expresses mutant Tau-P301S in neurons^10^. This leads to the accumulation of hyperphosphorylated filamentous tau, driving progressive neurodegeneration in the spinal cord and upper cortical layers, with physical deterioration of the mouse from around 5 months^10–12^. As such it represents a useful model of neurodegenerative tauopathy, although does not exhibit the pattern of neuronal loss found in AD. The APPswe/PS1dE9 mice^13^ (APP/PS1) ß-amyloidopathy mouse model exhibits extracellular Aß deposition, beginning at around 6 months, and increasing to 12 months^14^. Although neurodegeneration is modest, there is progressive cognitive decline^15^. Changes to astrocytes, assayed by GFAP expression/immunoreactivity, follow a comparable trajectory to neurodegeneration in the MAPT^P301S^ mouse^10–12^, and in the APP/PS1 mouse is evident from around 7–9 months^16^.

**Fig. 1:**
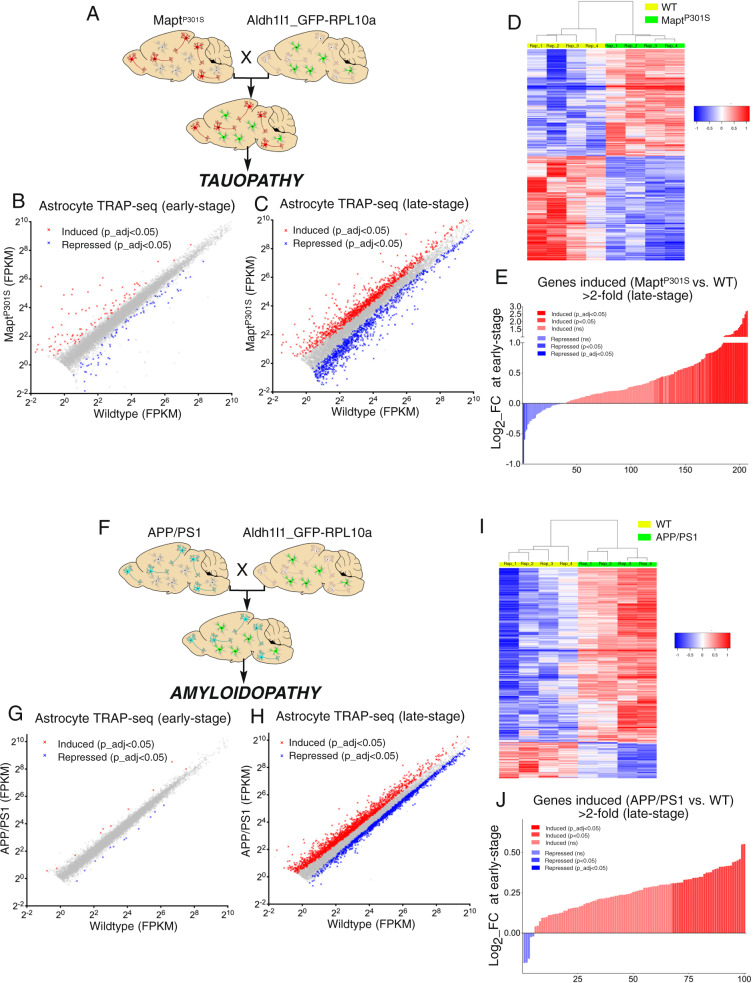
Changes to the astrocyte translatome due to tau and amyloid pathology. **A** Schematic illustrating the crossing of MAPT^P301S^ with the Aldh1l1_eGFP-RPL10a mouse. Astrocyte TRAP-seq performed on MAPT^P301S^ vs. WT mice (both carrying the Aldh1l1_eGFP-RPL10a allele) at 3 months (**B**) and 5 months (**C**) in the spinal cord. Genes significantly induced (red) and repressed (blue) are highlighted (expression cut-off 1FPKM, *p* values are adjusted for multiple testing by the Benjamini–Hochberg procedure to give a false discovery rate of 5% (p_adj < 0.05)) here and in all RNA-seq analyses; *n* = 4 mice per genotype. Genes in grey are not significantly changed. See also Supplementary Data 1 and 2. Note that all tests throughout the manuscript are 2-sided. **D** Sample-by-sample heat map of genes induced (red) or repressed (blue) >1.5-fold at 5 months (p_adj < 0.05). **E** Log2-fold change of the genes induced > 2-fold in MAPT^P301S^ at late stage (**C**) when examined at the early stage (**B**). *t* = 11.28, df = 206, p < 1E−15 (Ratio paired *t*-test of FPKM (WT) vs FPKM (MAPT^P301S^)). **F** Schematic illustrating the crossing of APP/PS1 mouse with the Aldh1l1_eGFP-RPL10a mouse. Astrocyte TRAP-seq performed on APP/PS1 vs. WT mice at 6 months (**G**) and 12 months (**H**) in the cortex. Genes significantly induced (red) and repressed (blue) are highlighted (expression cut-off 1FPKM, p_adj < 0.05); *n* = 4 mice per genotype. See Supplementary Data 3 and 4. **I** Sample-by-sample heat map of genes induced (red) or repressed (blue) >1.5 fold (p_adj<0.05) at 12 months. **J** Log2-fold change of the genes induced >2-fold in APP/PS1 at late stage (**H**) when examined at the early stage (**G**). *t* = 14.68, df = 100, *p* < 1E−15 (Ratio paired t-test of FPKM (WT) vs FPKM (APP/PS1)).

To define changes to astrocytic gene expression in response to amyloid or tau pathology, we performed translating-ribosome-affinity purification (TRAP) on astrocytes, by crossing the MAPT^P301S^ and APP/PS1 mice onto the TRAP line Aldh1l1_eGFP-RPL10a (Fig. 1A), which expresses GFP-tagged ribosomes specifically in astrocytes^17^, showing co-localisation with astrocyte markers Aldh1l1 and GFAP, but not neuronal or microglial markers (Supplementary Fig. 1A, B). Additionally, we verified that immunoprecipitation of ribosomes pulled down mRNA enriched in astrocyte-specific transcripts but not other cell-type-specific transcripts (Supplementary Fig. 1C). We then used TRAP-seq to profile changes to astrocytes in the MAPT^P301S^ mouse at early and late stages (3 and 5 months, spinal cord and cortex), and the APP/PS1 mouse at early and late stages (6 and 12 months, cortex), with these time periods and CNS regions reflecting the progression of astrogliosis in these models. TRAP-seq revealed modest numbers of significant gene changes in the astrocyte translatome at 3 months in the spinal cord in MAPT^P301S^ mice, but changes were substantial by 5 months (Fig. 1A–D, Supplementary Data 1 and 2). In the cortex, where degeneration only takes place in upper layers, numbers of significantly changed genes were lower, although, the fold-change (up and down) correlated well with the spinal cord data (Supplementary Fig. 1D, *r* = 0.77). This is indicative of qualitatively similar responses of cortical and spinal astrocytes to tau pathology, albeit with a weaker cortical response (linear regression slope: 0.33; 95% CI: 0.32–0.34), although region-specific responses cannot be ruled out.

We used the spinal cord data for subsequent enrichment analyses, since the larger numbers of significant genes provided greater power for these studies. With regard to the APP/PS1 mouse, few changes were observed in the astrocyte translatome in APP/PS1 mice at 6 months (Fig. 1G), with large changes evident by 12 months (Fig. 1H, I, Supplementary Data 3 and 4), consistent with the known progression of the pathology.

To determine whether gene expression changes evident in late-stage MAPT^P301S^ and APP/PS1 astrocytes were beginning to occur at the earlier stage, we took the groups of genes induced >2-fold at late stage and examined the fold change at the early stage in their respective models (Fig. 1E, J). This revealed that most of these genes showed a positive fold change in MAPT^P301S^ (Fig. 1E) and APP/PS1 (Fig. 1J) astrocytes at the early stage of disease, and the gene sets as a whole were significantly upregulated (Fig. 1E, J). This indicates that changes to astrocytes begin to occur early in both tauopathy and ß-amyloidopathy models, and become more pronounced later in disease.

### Tauopathy and ß-amyloidopathy exacerbate age-dependent reactive changes in astrocytes

**Fig. 2:**
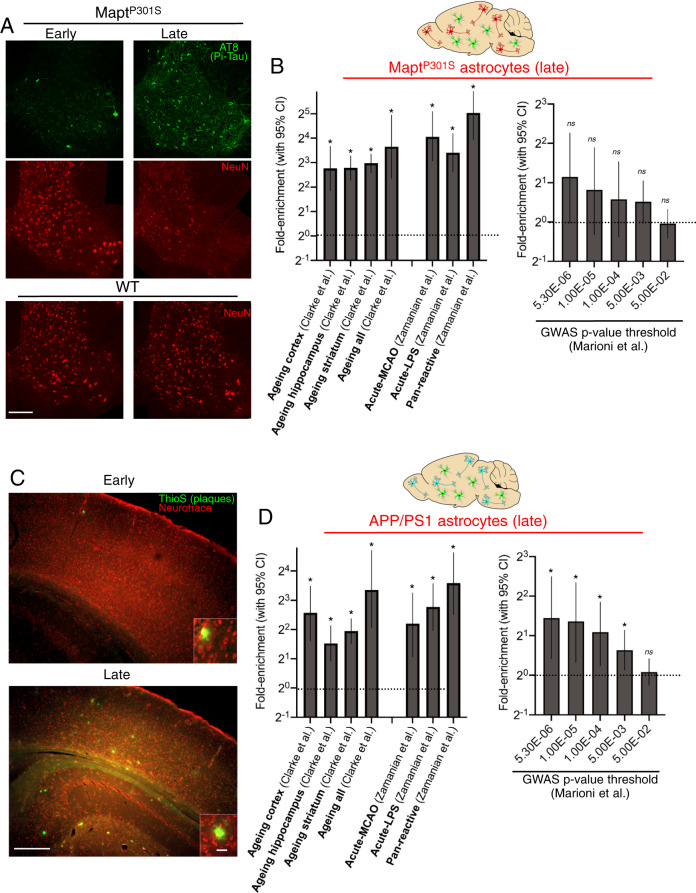
Comparison of astrocytes in tau and amyloid pathology with acutely induced reactive profiles, ageing astrocytes, and AD risk genes. **A** Example images of phospho-tau and NeuN staining of spinal cord sections at the indicated ages and genotypes, illustrating progressive accumulation of phospho-tau and neurodegeneration as previously reported^10^. Scale bar: 100 µm. **B** (Left) Genes induced >1.5 fold at 5 months in the MAPT^P301S^ mouse (expression cut-off 1FPKM, Benjamini–Hochberg p_adj<0.05) were taken and enrichment analysis performed on the indicated gene sets (see main text for details). Fold enrichment is shown, and 95% confidence interval (CI) of the fold enrichment depicted by the error bar. *p values (left-to-right, here and throughout the manuscript): 5.3E−07, 4.2E−22, 8.3E−42 1.7E−06, 2.6E−12, 5.6E−14, 1.2E−19 (two-sided Fisher’s exact test). **B** (right) Genes induced >1.5 fold at 5 months in the MAPT^P301S^ mouse (expression cut-off 1FPKM, Benjamini–Hochberg p_adj < 0.05) were taken and enrichment analysis performed on human genes of different *p* value cut-offs for association with late-onset AD^21^. *P* values: 0.082. 0.9. 0.29, 0.075, 0.90. **C** Example images of amyloid plaques (ThioS) and neurons (Neurotrace) staining of cortical sections at the indicated ages of the APP/PS1 mouse, illustrating progressive accumulation of plaques and lack of neurodegeneration as previously reported^91^. Scale bar: 300 µm (main picture); 20 µm (inset). **D** (left) Genes induced >1.5 fold at 12 months in the APP/PS1 mouse (expression cut-off 1FPKM, p_adj < 0.05) were taken and enrichment analysis performed exactly as in **B**  (left), with fold enrichment is shown, and 95% CI depicted by error bar. *p values: 4.1E−06, 6.0E−06, 3.5E−15, 2.0E−05, 0.0006, 3.0E−08, 3.1E−09 (two-sided Fisher’s exact test). **D** (right). Enrichment analysis of genes induced >1.5 fold at 12 months in the APP/PS1 mouse for human genes of different *p* value cut-offs for association with late-onset AD. *p values: 0.011, 0.028, 0.017, 0.020; 0.63 (ns) (two-sided Fisher’s exact test).

Since age is the major risk factor for AD, we next exploited a recently published data-set of the ageing astrocyte translatome (10 weeks vs 24 months^18^) to ask whether changes induced in MAPT^P301S^ and APP/PS1 astrocytes are enriched in those genes which change during normal ageing. We studied published gene sets that described genes induced >2-fold in the ageing cortex, hippocampus and striatum, as well as the set of genes common to all three regional sets. In all cases we observed an enrichment of age-dependent genes in the upregulated genes in both the MAPT^P301S^ and APP/PS1 TRAP-seq data (Fig. 2B left, Fig. 2D left). This strongly suggests that both Aß and Tau pathology prematurely induce signatures normally only found in the astrocytes of old mice.

While chronic neurodegeneration is associated with reactive astrogliosis, astrogliosis can also be induced very rapidly (<24 h) by acute insults such as inflammatory stimuli or stroke. We wanted to define whether changes in astrocytes by chronic Tau and Aß pathology bore resemblance to acutely induced reactive profiles. We used the data from Fig. 1, taken at a time when some neurodegeneration had taken place in the MAPT^P301S^ mouse^10,12^ (Fig. 2A), and significant plaque load had built up in the APP/PS1 mouse (Fig. 2C)^14^. Recent studies on astrocytes in neurodegenerative disease have contended that they more closely resemble a reactive profile induced by acute inflammation (LPS) rather than acute stroke (MCAO)^19^, although these conclusions were based on limited numbers of markers (see Discussion). Rather than use these markers, we performed an unbiased analysis of acute (24 h) LPS and MCAO transcriptome data^20^ (GSE accession number GSE35338) to create sets of genes preferentially induced by acute LPS, acute MCAO, and pan-reactive genes induced equally by both treatments (Supplementary Fig. 2A–C, Supplementary Data 5, see “Methods” for description of strategy). Both Tau and Aß pathology-induced changes were enriched in all three gene sets, consistent with a reactive phenotype in both disease models. However, we found no evidence that either Tau or Aß pathology induced an astrocyte profile more closely resembling a specific (LPS vs. MCAO) acute response.

### Astrocytes in tauopathy and ß-amyloidopathy mice show differential enrichment in AD risk genes

A recent advance in understanding human AD is the discovery of increased numbers of AD risk genes, many of which are expressed in astrocytes. We performed enrichment analysis of Tau and Aß-induced changes in astrocytes for AD risk genes and sub-threshold risk loci^21^, using an approach recently performed on microglia^22^, focussing on the 9938 genes expressed > 1FPKM in our TRAP-seq data for which a gene-level GWAS *p* value was available^21^, looking for enrichment in genes induced >1.5-fold (p_adj<0.05) within our data sets. Using a Bonferroni-corrected cut-off of 0.05/9938 (i.e. 5.03E−06), induced genes in the APP/PS1 TRAP-seq data were significantly enriched in AD risk genes (Fig. 2D, right). Moreover, sequential relaxing of the *p* value cut-offs to include more sub-threshold risk genes maintained significant enrichment down to *p* = 5E−03, with enrichment disappearing at the *p* = 5E−02 cut-off (Fig. 2D, right). In contrast, a similar analysis of genes induced in MAPT^P301S^ astrocytes did not achieve significance at any *p* value cut-off (Fig. 2B, right). This observation raises the possibility that astrocyte-centred genetic risk may influence their response to early Aß pathology, rather than Tau, as has been suggested for microglia^22^.

### A core signature of astrocytic genes regulated by both Tau and Aß pathology

**Fig. 3:**
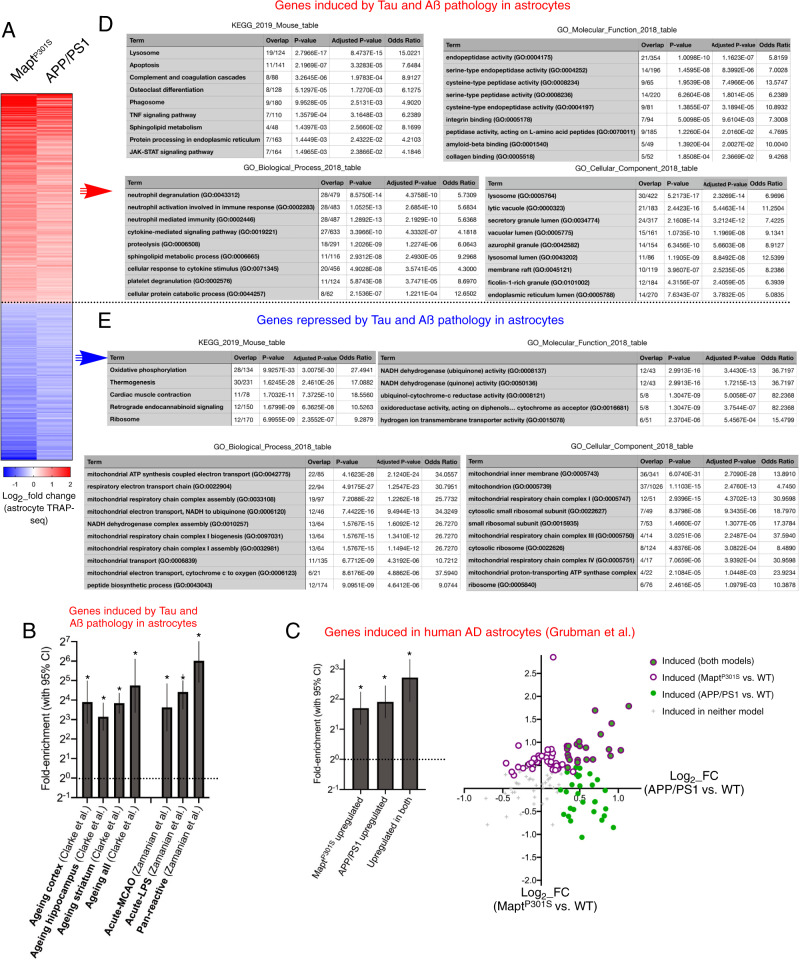
Tau and amyloid pathology trigger a core set of gene expression changes in astrocytes. **A** A heat map of genes induced (red) and repressed (blue) in both MAPT^P301S^ and APP/PS1 mice. **B** Genes induced in astrocytes in both MAPT^P301S^ and APP/PS1 mice (p_adj < 0.05 in both sample sets) were subjected to enrichment analysis against the indicated gene sets. *3.3E−07, 4.6E−12, 1.9E−34, 6.4E−07, 2.0E−05, 3.1E−13, 2.4E−19 (two-sided Fisher’s exact test). Fold enrichment and 95% CI shown. **C** (Left) Enrichment analysis of genes induced in human AD astrocytes^7^ for which a 1:1 ortholog exists (and >10 FPKM cut-off) for genes induced in MAPT^P301S^ astrocytes (Fig. 1C), APP/PS1 astrocytes (Fig. 1H), or those induced in both models (**A**). Fold enrichment and 95% CI shown, *p = 9.4E−05, 4.1E−11, 4.0E−08 (two-sided Fisher’s exact test). (Right) For genes induced in human AD astrocytes^7^ for which a 1:1 ortholog exists and that are expressed >10 FPKM in MAPT^P301S^ and APP/PS1 astrocytes, the Log(2) fold change in each gene is shown for both models (y-axis: MAPT^P301S^ vs. WT; *x*-axis: APP/PS1 vs. WT). For each gene, in indication of whether it is significantly (*p* < 0.05) induced in either, both, or neither models is indicated. Ontological analysis of genes induced (**D**) or repressed (**E**) in astrocytes in both MAPT^P301S^ and APP/PS1 mice. For KEGG pathway analysis, disease pathways were omitted (to enable a focus on biological pathways) and the number of genes required was ≥5. For KEGG and GO ontology analysis the top ten pathways are shown, unless fewer than ten achieved an adjusted *p* value cut-off of 0.05.

Despite the difference noted above, the sets of genes altered in MAPT^P301S^ and APP/PS1 astrocytes showed significant overlap (both induced and repressed genes, *p* < 0.0001 (Fisher’s exact test)), with a ‘core’ set of 203 genes significantly upregulated in both MAPT^P301S^ and APP/PS1 models, and a core set of 151 genes downregulated (Fig. 3A, Fig, S3A, Supplementary Data 6). As expected, the core upregulated gene set was strongly enriched in age-dependent genes and acutely induced reactive astrocyte genes (Fig. 3B).

Recent post-mortem transcriptomic studies have begun to shed light on the state of astrocytes in human AD. We wanted to compare the astrocyte responses to Aß and Tau pathology (our study) to post-mortem data sets in which both pathologies are evident. We used data from a recent single nucleus RNA-seq study^7^ which identified genes induced in astrocytes in AD relative to controls. We performed enrichment analysis on those genes with 1:1 orthologs in mouse, expressed >10 FPKM (a higher than normal threshold since highly expressed genes are more likely to be detected in single-cell RNA-seq), and observed that genes upregulated in the translatome of both MAPT^P301S^ and APP/PS1 astrocytes were enriched in genes induced in human AD astrocytes and moreover, the core set of genes induced by both Tau and Aß pathology was also enriched (Fig. 3C, left). Of 126 genes expressed >10 FPKM in either or both of MAPT^P301S^ and APP/PS1 astrocytes, 68/126 (p_adj < 0.05) or 86/126 (*p* < 0.05) were induced by Aß pathology, Tau pathology, or both (Fig. 3C, right). Thus, there is significant association of genes induced in astrocytes by Tau and Aß pathology in mice over weeks/months, and those elevated in human astrocytes post-mortem after many years of patho-progression. Of note, however, a relatively small number of AD patients were studied by Grubman et al. (six), and, while a significant enrichment is observed (Fig. 3C), as larger human data sets become available the extent of the enrichment will become clearer.

We next performed ontological analysis of the core set of genes upregulated by both Tau and Aß pathology which revealed that GO Biological Processes were dominated by those associated with cytokine and inflammatory responses, as well as protein degradation (Fig. 3D). GO Molecular Functions were dominated by proteases, peptidases and protein binding classes, while GO Cellular Components included lysosomal and luminal compartment of several types (Fig. 3D). High-ranking KEGG pathways included Lysosome (Fig. 3D, Supplementary Fig. 3B), Apoptosis, plus a number of inflammation-associated pathways (Fig. 3D). Studying the genes repressed in both MAPT^P301S^ and APP/PS1 models, GO Biological Processes were dominated by mitochondrial oxidative phosphorylation and general protein synthesis, GO Molecular Functions were by mitochondrial enzymic activities, and GO Cellular Components included mitochondrial protein classes and subclasses, as well as ribosomal proteins (Fig. 3E). Important KEGG pathways enriched in the downregulated genes included oxidative phosphorylation (Fig. 3E, Supplementary Fig. 3C) and ribosome (Fig. 3E), particularly the large subunit (*Rpls12, 15a, 18, 21, 27, 27l, 28*, and *41*). Collectively, these analyses suggest that both Tau and Aß induce potentially harmful alterations to astrocytes, such as mitochondrial and protein synthesis deficits. However, the induction of genes within pathways associated with protein degradation and clearance may represent a beneficial/protective response of astrocytes to proteinopathy.

### Nrf2 targets form part of the core signature of astrocytic genes regulated by Tau and Aß pathology

**Fig. 4:**
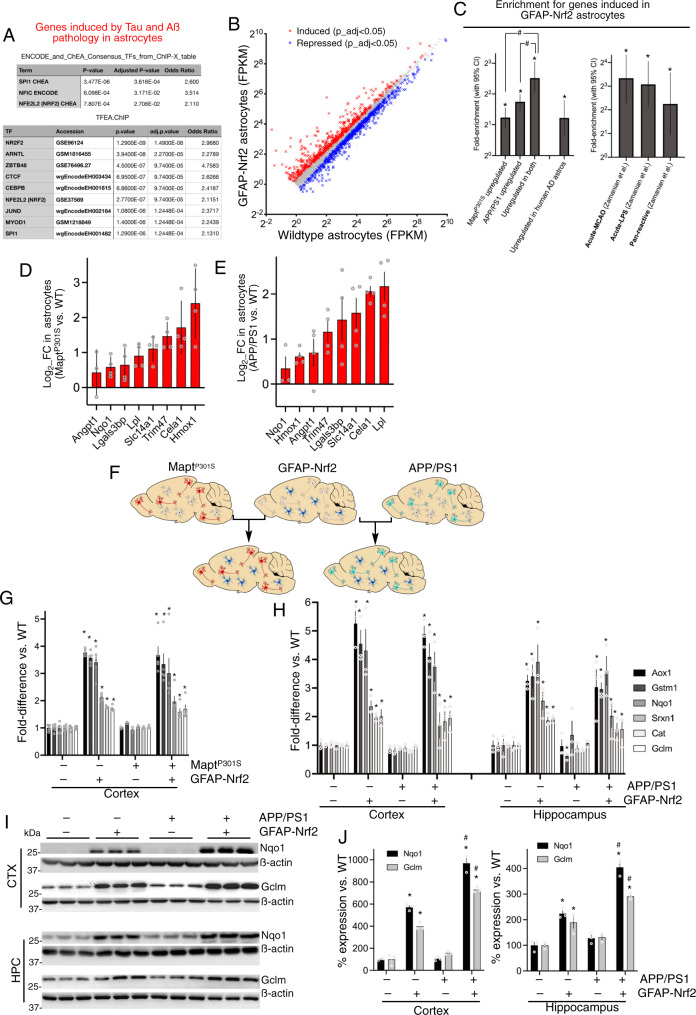
Activating Nrf2 in models of tauopathy and ß-amyloidopathy. **A** Enrichment analysis of genes induced in MAPT^P301S^ and APP/PS1 mice for transcription factor (TF) targets performed in Enrichr^23^ (upper) and TFEA.ChiP^24^ (lower). **B** RNA-seq analysis of astrocytes sorted from GFAP-Nrf2 mice, vs. WT (*n* = 5). Genes induced (red) and repressed (blue) are highlighted (>1FPKM, Benjamini–Hochberg-adjusted *p* value (p_adj) <0.05). **C** (Left). Enrichment of genes induced in GFAP-Nrf2 astrocytes for genes induced in MAPT^P301S^ astrocytes (Fig. 1C), APP/PS1 astrocytes (Fig. 1H), and in both (Fig. 3A), and for genes induced in human AD astrocytes^7^. *p values: 4.23E−11, 4.1E−21, 8.48E−15, 0.0001 (Fisher’s exact test). ^#^*p* values: 0.0008, 0.013 (normal approximation to difference in log odds ratios). (Right) Enrichment of genes induced in GFAP-Nrf2 astrocytes for gene sets in Supplementary Fig. 2A–C. *p values: 8.7E−07, 4.0E−05, 0.007 (Fisher’s exact test). qPCR analysis of the indicated genes in MAPT^P301S^ and APP/PS1 astrocyte translatome. Two-way ANOVA for genotype effect: *F* (1,53) = 23.29, *p* = 1.2E−05 (**D**); *F* (1,55) = 46.13, *p* = 8.3E−09 (**E**) (*n* = 4). **F** Illustrating our crossing strategy. **G** Expression of selected Nrf2 target genes in the cortex (from RNA-seq). *p_adj: 1.7E−178, 2.5E−119, 9.4E−45, 2.3E−64, 3.9E−59, 4.1E−54, 8.6671E−61, 2.52E−22, 1.2E−14, 2.6E−15, 7.3E−11, 1.2E−11. **H** Analysis repeated on hippocampal tissue. *p_adj: 4.3E−31, 3.9E−27, 2.3E−12, 1.9E−07, 8.3E−07, 0.0018, 1.6E−33, 5.7E−45, 4.1E−14, 1.6E−06, 8.9E−10, 6.1E−10; adjusted *p* values (hippocampus): 3.8E−11, 8.2E−14, 1.0E−06, 3.2E−08, 0.0012, 1.03E−05, 0.049, 7.5E−09, 1.6E−11, 2.9E−08, 1.6E−09, 0.049, 0.007 (*n* = 3). **I**, **J** Western analysis of cortical and hippocampal Nqo1 and Gclm. Two-way ANOVA for cortical Gclm: *F* (1, 8) = 98.68, *p* = 8.9E−06 (APP/PS1 effect); F (1, 8) = 493.2; *p* = 1.79E−08 (GFAP-Nrf2 effect); two-way ANOVA for cortical Nqo1: *F* (1, 8) = 69.4, *p* = 3.3E−05 (APP/PS1 effect); *F* (1, 8) = 773.2 *p* = 3.0E−09 (GFAP-Nrf2 effect). Two-way ANOVA for hippocampal Gclm: *F* (1, 8) = 26.67, *p* = 0.0009 (APP/PS1 effect); *F* (1, 8) = 91.36, *p* = 1.2E−05 (GFAP-Nrf2 effect); 2-way ANOVA for hippocampal Nqo1: *F* (1, 8) = 59.51, *p* = 5.7E−05 (APP/PS1 effect); *F* (1, 8) = 219.1, *p* = 4.3E−07 (GFAP-Nrf2 effect), *n* = 3 mice. *p values for GFAP-Nrf2 effect: 1.4E−06, 9.8E−06, 3.7E−08, 3.8E−04, 0.0025, 1.0E−06, 4.9E−08 (Bonferroni’s post-hoc test). ^#^*p* values for APP/PS1 effect: 5.3E−06, 4.4E−06, 2.6E−05, 0.0011.

With regard to putative mediators of the co-induced genes, a survey of ENCODE and ChEA Consensus target genes from ChIP-X on the Enrichr platform^23^ revealed that upregulated genes were enriched in known mediators of the effects of inflammatory cytokines SPI1, plus NFIC, and also NFE2L2/Nrf2 (Fig. 4A, upper). Of these three transcription factors, ChIP-seq data sets of two (SPI1 and NFE2L2/Nrf2) were also enriched upon interrogation of this gene set on the TFEA.ChiP platform^24^ (Fig. 4A, lower-highest ranked factors shown). Nrf2 is a master regulator of antioxidant, detoxification, and proteostasis genes^25–27^, raising the possibility that this pathway may represent an adaptive-protective response to Aß and Tau pathology by astrocytes. To further determine whether Aß/Tau-induced astrocytic genes contain an element of Nrf2-dependent gene expression, we first defined the set of genes downstream of Nrf2 activation in astrocytes by performing RNA-seq on astrocytes sorted (by MACS) from the cortices of transgenic mice which overexpress Nrf2 (approximately threefold) specifically in astrocytes^28^ (GFAP-Nrf2), (Fig. 4B, Supplementary Data 7). We observed that of the core Aß/Tau-induced astrocytic genes, 29% were also induced in GFAP-Nrf2 astrocytes (*p* < 0.05) and 21% (p_adj < 0.05), a near sixfold enrichment over chance (Fig. 4C, left) and a stronger enrichment than either Aß-induced or tau-induced gene sets separately (Fig. 4C, left). This is suggestive that Nrf2 is indeed part of a core response of astrocytes to Aß and Tau pathology and, consistent with this, genes induced in GFAP-Nrf2 astrocytes were also found to be enriched in genes upregulated in human AD astrocytes (Fig. 4C, left).

For eight genes induced in both models as well as in GFAP-Nrf2 astrocytes, for which there is published data that they are direct Nrf2 target genes^29–36^ we confirmed their up-regulation in the MAPT^P301S^ and APP/PS1 astrocyte translatome by qPCR (Fig. 4D, E). We also confirmed induction of Nrf2 target Hmox1 in astrocytes in the MAPT^301S^ and APP/PS1 astrocytes by histological methods (Supplementary Fig. 4B, C). Additionally, we took genes induced >2-fold in GFAP-Nrf2 astrocytes and assessed the number significantly regulated (up or down) in the MAPT^P^^301S^ and APP/PS1 astrocyte translatome data sets. Twenty-one genes induced >2-fold in GFAP-Nrf2 astrocytes are also significantly regulated in the MAPT^P^^301S^ astrocyte translatome (16 up and 6 down, Supplementary Fig. 4D). Eighteen genes induced >2-fold in GFAP-Nrf2 astrocytes are also significantly regulated in the APP/PS1 astrocyte translatome (15 up and 3 down, Supplementary Fig. 4E). For both models this reflects a significant up-regulation of the set (two-way ANOVA).

Also of note, the subset of Aß/Tau-induced astrocytic genes that were also induced in GFAP-Nrf2 astrocytes were found to be enriched in Nrf2 target genes (as expected), and also in KEGG/GO lysosomal and protease functions and pathways (Supplementary Fig. 4A). This suggests that Nrf2 may contribute to these signatures in astrocytes from the MAPT^P301S^ and APP/PS1 astrocytes. Since both amyloidopathies and tauopathies are disorders of protein aggregation and disrupted proteostasis, we reasoned that Nrf2 may drive an adaptive-protective signature in reactive astrocytes. For example, Nrf2 activation in astrocytes may influence protein degradation pathways to boost astrocyte-mediated clearance of toxic protein moieties, and Nrf2-dependent antioxidant pathways may promote non-cell-autonomous neuroprotection and immunomodulation by enhancing brain redox homoeostasis. Analysis of all genes induced in GFAP-Nrf2 astrocytes showed strong enrichment for both ‘pan-reactive’ gene set, and similar enrichment for both ‘acute LPS’ and ‘acute MCAO’ gene sets (Fig. 4C, right). Thus, by these criteria Nrf2 activation is sufficient to induce a ‘reactive’ transcriptional profile and, given the cytoprotective signatures induced in GFAP-Nrf2 astrocytes, we set about determining whether they could alter disease trajectory. To do this we crossed the GFAP-Nrf2 mouse onto both MAPT^P301S^ and APP/PS1 mouse lines (Fig. 4F). Bulk RNA-seq of both the cortex and hippocampus of GFAP-Nrf2 mice revealed an increase in expression of a number of known Nrf2 target genes, including *Aox1*, *Gstm1*, *Srxn1*, *Cat*, *Nqo1* and *Gclm* (Fig. 4G, H). Importantly, the Nrf2 pathway remained active in both the MAPT^P301S^ and APP/PS1 mice: these target genes were also elevated in the MAPT^P301S^_X_GFAP-Nrf2 and APP/PS1_X_GFAP-Nrf2 mice, compared to MAPT^P301S^ and APP/PS1 respectively (Fig. 4G, H), confirmed at the protein level for Gclm and Nqo1. Moreover, the APP/PS1 genotype enhanced Nqo1 and Gclm expression against the GFAP-Nrf2 background (Fig. 4J), suggestive of synergistic effects on Nrf2 target gene expression (overexpression of Nrf2 (GFAP-Nrf2) combined with Nrf2 activating pathology (APP/PS1)).

We took steps to rule out expression of the GFAP-Nrf2 transgene in neurons. Firstly, we generated primary cultures of astrocytes, and of (astrocyte-free) neurons using techniques described by us before^37^ (Supplementary Fig. 4F). This takes advantage of the fact that expression of Nrf2 in cortical neurons is extremely low, and so by studying them in isolation we would be able to detect even modest GFAP-Nrf2 transgene. Nrf2 expression in immature (DIV 2) cortical neurons is low (approximately 8% of that in astrocytes) and drops further as they mature to DIV 10 (2% of that in astrocytes), due to epigenetic repression of the endogenous Nrf2 gene promoter^37^. Examination of Nrf2 levels in cells derived from GFAP-Nrf2 mice revealed that while Nrf2 levels in astrocytes were 2-fold higher, those in neurons were unchanged (Supplementary Fig. 4G). We see qualitatively the same thing when studying the Nrf2 target gene Nqo1 (Fig. S4H). Therefore, these data do not support the notion of neuronal leakage of the GFAP-Nrf2 transgene. Additionally, we performed RNAscope for Nrf2 in WT vs GFAP-Nrf2 mice brains. Neurons were identified based on anatomical location and according to established neuropathological criteria including size and morphology. Neuronal cells have a distinct morphology compared to glial cells. Neuronal cytoplasm stains with hematoxylin counterstain and the diameter of their nuclear membrane is larger. Hematoxylin stains only the nuclei of glial cells and not their processes. In the WT cortex Nrf2 transcripts were distributed diffusely in the parenchyma and absent from neuronal cell bodies, consistent with low basal neuronal expression of Nrf2 (Supplementary Fig. 4I). In the GFAP-Nrf2 mouse an increase in transcripts was visualised proximal to glial nuclei but not within the cell bodies of neurons (Supplementary Fig. 4I). We also ruled out expression of the GFAP-Nrf2 transgene in microglia, which were sorted by MACS, leading to high enrichment in expression of microglial marker *Cx3cr1*, and depletion of astrocyte marker *Gfap*. The microglia isolated from GFAP-Nrf2 mice showed no change in levels of *Nrf2* nor Nrf2 target gene *Nqo1* (Supplementary Fig. 4K), thus further confirming the astrocyte specificity of Nrf2 in the GFAP-Nrf2 line.

### Astrocytic Nrf2 slows tauopathy progression

**Fig. 5:**
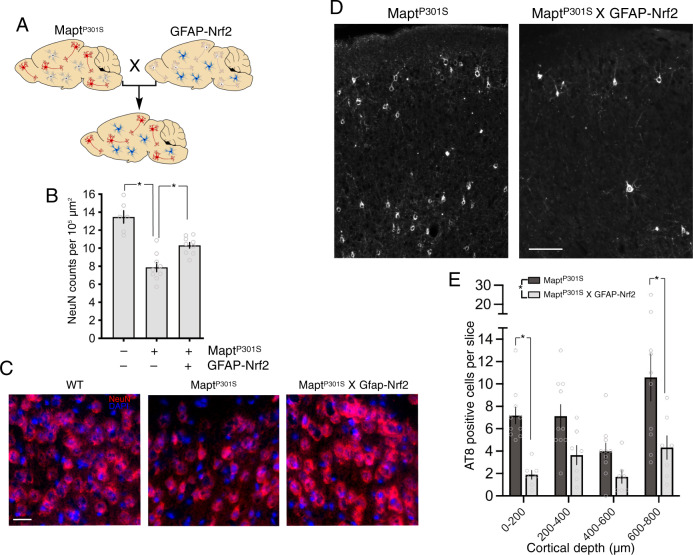
Astrocytic Nrf2 reduces phospho-tau accumulation and neurodegeneration. **A** Schematic illustrating the crossing of MAPT^P301S^ and GFAP-Nrf2 mice. **B**, **C** NeuN staining of upper cortical layers of mice at early-stage disease. Mean ± SEM shown. *F* (2, 21) = 28.83, *p* = 9.5E−7 (1-way ANOVA); * Post-hoc (Bonferroni) *p* values: 3.8E−07, 0.0028. *N* = 6 WT, 9 MAPT^P301S^ and 9 MAPT^P301S^_X_GFAP-Nrf2 mice. Example pictures shown in (**C**); scale bar 20 µm. **D**, **E** Cortical slices were subject to AT8 immunofluorescent staining, and positive cells counted. Mean ± SEM shown from *n* = 10 MAPT^P301S^ and *n* = 7 MAPT^P301S^_X_GFAP-Nrf2 mice. Where more than one slice was analysed per mouse, and average was taken to provide a single data point per mouse. Main genotype effect: *F* (1, 60) = 26.54, *p* = 3.02E−6 (two-way ANOVA). Post-hoc (Bonferroni) *p* values: 0.010, 0.0017. **D** Shows example pictures, scale bar 100 µm.

Focussing first on the MAPT^P301S^_X_GFAP-Nrf2 mouse (Fig. 5A), we found that it exhibited reduced cortical neurodegeneration compared to the MAPT^P301S^ mouse (Fig. 5B, C), indicative of a non-cell-autonomous neuroprotective effect of Nrf2-driven reactive astrocytes. Cortical neurodegeneration in this MAPT^P301S^ mouse model is not associated with cortical atrophy at this stage (^11^, confirmed in Supplementary Fig. 5A). As a control we analysed human MAPT^P301S^ expression in the MAPT^P301S^ and MAPT^P301S^_X_GFAP-Nrf2 mice (using species-specific RNA-seq read separation^38^) and found no influence of the GFAP-Nrf2 transgene (Supplementary Fig. 5B), ruling out repression of the transgene as the mechanism of protection (mouse *Mapt* levels were also unaffected, Supplementary Fig. 5B). We next quantified levels of AT8-positive phospho-tau accumulation, and found them to be lower in MAPT^P301S^_X_GFAP-Nrf2 compared to the MAPT^P301S^ mice (Fig. 5D, E), indicative of a pro-clearance or anti-aggregation/phosphorylation effect of astrocytic Nrf2 activation.

Reduction of neurodegeneration in the spinal cord was also observed (Supplementary Fig. 5C), along with amelioration of loss of expression of neurofilament NF200, and synaptic marker PSD-95, There was also inhibition of reactive astrocyte marker Gfap expression (Supplementary Fig. 5D, E), although Iba1 induction was not reduced (Supplementary Fig. D, F). Of note, while Nrf2-driven gene expression can protect cells against oxidative stress (both directly and non-cell-autonomously), we did not observe a reduction in reduced glutathione levels (a measure of oxidative stress) in MAPT^P301S^ tissue (Supplementary Fig. 5G). This suggests that the protective effect of astrocytic Nrf2 on Tau pathology may not be as simple as rectifying global redox imbalance.

**Fig. 6:**
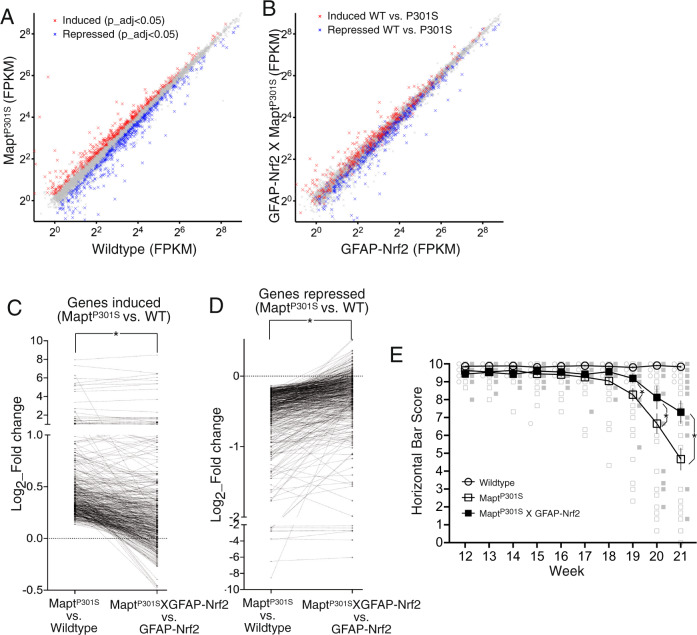
Astrocytic Nrf2 slows transcriptional perturbation and physical deterioration of the MAPT^P301S^ mouse. **A** RNA-seq analysis of the early-stage neocortex (MAPT^P301S^ vs WT). Genes induced (red) and repressed (blue) are highlighted (>1FPKM, Benjamini–Hochberg-adjusted p value (p_adj)<0.05); *n* = 4 animals per genotype. **B** RNA-seq analysis of early-stage neocortex (MAPT^P301S^_X_GFAP-Nrf2 vs. GFAP-Nrf2). Genes induced (red) and repressed (blue) from **A**  are highlighted; *n* = 4 animals per genotype. **C**, **D** A comparison of the fold change in gene expression caused by MAPT^P301S^ expression in an otherwise WT background (i.e. MAPT^P301S^ vs. WT) compared to the fold change in gene expression caused by MAPT^P301S^ expression against a GFAP-Nrf2 background (i.e. MAPT^P301S^ vs. MAPT^P301S^_X_GFAP-Nrf2). Genes induced (**C**) or repressed (**D**) (i.e., genes highlighted in **B**) are shown. **C** Note how GFAP-Nrf2 represses the induction of MAPT^P301S^ -induced genes and inhibits the repression of MAPT^P301S^ -repressed genes. *t* = 11.12, df = 398, *p* = 1.7E−25 (**C**); *t* = 11.89, df = 458, *p* = 1.2E−28 (**D**), paired *t*-test. **E** Horizontal bar performance of mice of the indicated genotypes. Mean ± SEM shown (*n* = 39 WT, 53 MAPT^P301S^, 17 MAPT^P301S^_X_GFAP-Nrf2 mice studied at 19 and 20 weeks; 32 WT, 35 MAPT^P301S^, 17 MAPT^P301S^_X_GFAP-Nrf2 at 21 weeks). Two-way ANOVA reveals a main genotype effect (*F* (2, 83) = 23.13, *p* = 1.04E−8). *Bonferroni’s post-hoc test (left to right): *p* = 0.0338, 0.00028, 2.93E−11.

To further probe this protective effect we identified genes up- and downregulated globally in the bulk tissue of the neocortex of the MAPT^P301S^ mouse (by RNA-Seq) in an otherwise wild-type background (WT vs. MAPT^P301S^, Fig. 6A, Supplementary Data 6). We then assessed the perturbation of these genes by MAPT^P301S^ in a GFAP-Nrf2 background (i.e. GFAP-Nrf2 vs. MAPT^P301S^_X_GFAP-Nrf2, Fig. 6B). We found that astrocytic Nrf2 overexpression substantially rescued cortical transcriptomic perturbation in brain tissue driven by the MAPT^P301S^ transgene: the clear separation of MAPT^P301S^-induced (red) and repressed (blue) genes in Fig. 6A is largely absent in Fig. 6B. This rescue is illustrated by the fact that MAPT^P301S^-induced genes are overall repressed by astrocyte Nrf2 expression (Supplementary Fig. 6A, upper) and MAPT^P301S^ -repressed genes are overall enhanced by astrocyte Nrf2 expression (Supplementary Fig. 6A, lower). Consistent with this analysis, fold-induction of MAPT^P301S^ -induced genes and fold-repression of MAPT^P301S^-repressed genes are both far lower in mice overexpressing Nrf2 in astrocytes (Fig. 6C and D). Of note, basal expression of MAPT^P301S^-induced and MAPT^P301S^-repressed genes are largely unaffected in GFAP-Nrf2 mice (Supplementary Fig. 6B), ruling out changes in the basal ‘denominator’ as a cause of difference in fold change. Thus, astrocytic Nrf2 represses the appearance of molecular and pathological features of tauopathy in the MAPT^P301S^ mouse. As noted above, MAPT^P301S^ mouse suffers from progressive neurodegeneration and as a result the mouse deteriorates physically, measurable by horizontal bar performance. Consistent with the molecular and cellular rescue effects reported above, we observed that astrocytic Nrf2 delayed the decline of horizontal bar performance, indicative of attenuated physical deterioration of the mouse (Fig. 6E).

### Astrocytic Nrf2 slows ß-amyloidopathy progression

**Fig. 7:**
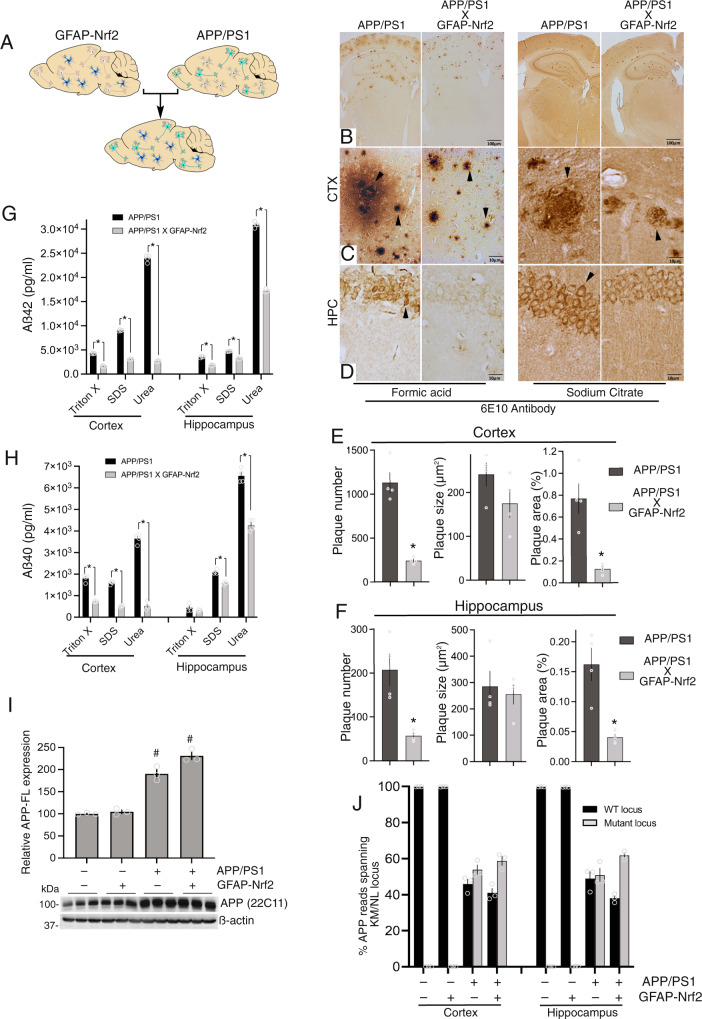
Astrocytic Nrf2 reduces Aß pathology. **A** Schematic illustrating the crossing of APP/PS1 and GFAP-Nrf2 mice. **B**–**D** Brains were fixed in 4% of paraformaldehyde and embedded in paraffin. The brains were cut into 10 μm coronal sections. Antigen retrieval was performed using either formic acid or sodium citrate buffer prior to immunohistochemical staining with 6E10 antibody and processed by VECTASTAIN^®^ Elite^®^ ABC kit. (**B**) shows an example picture at low magnification, with (**C**) and (**D**) focusing on the cortex and hippocampus (CA1), respectively. Arrowheads indicate 6E10 staining in plaques and neurons of APP/PS1 and APP/PS1_X_GFAP-Nrf2 mice. Quantification of total plaque number (left), average diameter (middle) and percent area covered (right) in the cortex (**E**) and hippocampus (**F**). Mean ± SEM shown here and throughout this figure. *p = 0.0003, 0.0033 (**E**); 0.0015, 0.0047 (**F**), unpaired two-sided t-test (*n* = 4 per mice per genotype). **G**, **H** The level of human Aß42 (**E**) and Aß40 (**F**) in Triton-X, SDS, and urea fractions in cortex and hippocampus was quantified by sandwich ELISA. Two-way ANOVA (main genotype effect) for Aβ42: *F* (1, 18) = 6127, *p* < 2.9E−24 (cortex); *F* (1, 18) = 2357, *p* < 1.5E−20 (hippocampus). *p values for (**G**): 7.1E−09, 6.8E−09, 9.7E−17, 0.003, 4.2E−12 (Bonferroni post-hoc test). Two-way ANOVA (main genotype effect) for Aβ40: *F* (1, 18) = 161.2, *p* = 4.9E−17 (cortex); *F* (1, 18) = 161.2, *p* = 2.0E−10 (hippocampus). *p values for (H): 1.2E−09, 1.4E−15, 2.1E−25, 1.9E−07, 1.5E−06, 1.20E−22 (Bonferroni post-hoc test). *N* = 4 animals per genotype. **I** Western analysis of whole cortical extracts for levels of full-length APP, using the 22C11 antibody. One-way ANOVA *F* (3, 8) = 99.92, *p* = 1.1E−06 followed by Bonferroni’s post-hoc test (*n* = 3 mice per genotype). *p < 5.6E−05, 3.3E−06, compared to WT. **J** RNA-seq reads from both cortex and hippocampus spanning the KM/NL Swedish locus across all four genotypes were scored as WT or mutant, and the % calculated (*n* = 3 mice per genotype).

We next looked at the influence of Nrf2-overexpressing astrocytes on the APP/PS1 mouse (Fig. 7A). Immunohistochemical analysis (6E10 antibody) of the brain revealed reduced levels of APP/Aβ staining in APP/PS1_X_GFAP-Nrf2 mice compared to APP/PS1 mice (Fig. 7B, both formic acid and sodium citrate treated slices). Astrocytic Nrf2 reduced plaque density in the cortex and hippocampus (Fig. 7C–F, Supplementary Fig. 7A) and also intracellular staining (most clearly seen in the CA1 region, Fig. 7D). Note though that this staining will be partly due to human full-length APP, as well as Aß. The 6E10 antibody preferentially recognises human over mouse protein (e.g. see^39^) since the human epitope it detects is not fully conserved in mouse APP. Total plaque number, average plaque size, and percent plaque area in cortex and hippocampus were determined (Fig. 7E, F). The number and area were significantly reduced in both brain regions of the APP/PS1_X_GFAP-Nrf2 compared to APP/PS1.

When present in plaques or intracellular inclusions, Aβ is resistant to extraction by conventional non-ionic detergents, requiring SDS or, *in extremis*, urea. To quantify the influence of astrocytic Nrf2 on Aβ presence in plaques and inclusions, Aß40 and Aß42 were quantified in APP/PS1 and APP/PS1_X_GFAP-Nrf2 brains by ELISA making sequential fractions of proteins soluble in denaturing agents of increasing stringency: Triton-X-100, SDS, and urea. This analysis revealed the existence of most Aß42, and around half of Aß40, in the Triton-insoluble/SDS-insoluble/urea-denatured fraction. Importantly, levels of Aß40 and Aß42 were reduced in both SDS and urea extracts analysed in APP/PS1_X_GFAP-Nrf2 mice relative to APP/PS1 mice (Fig. 7G, H).

These ELISA assays were complemented with western blots of similarly created fractions. Two primary bands were noted, representing full-length APP (APP-FL) and Aβ (Supplementary Fig. 7B, C). We observed a significant reduction in band intensity of APP-FL in both SDS and urea fractions, comparing APP/PS1_X_GFAP-Nrf2 to APP/PS1 (Supplementary Fig. 7B–D). Although APP-FL is not responsible for forming plaques, APP-FL-containing neuritic dystrophies is known to accumulate within plaques in human AD and models thereof^40,41^, something we also observe (using the 22C11 APP antibody which detects aa 66-81 of APP i.e. not Aß, Supplementary Fig. 7E). Thus, presence of APP-FL in SDS and urea fractions, and its reduction in these fractions in the APP/PS1_X_GFAP-Nrf2 mouse (where plaque load is reduced) is to be expected. In contrast, APP-FL levels in the Triton-soluble fraction were minimally affected, suggesting that astrocytic Nrf2 was preferentially reducing accumulation in plaques and other aggregates, rather than reducing overall expression of APP. To look into this further, we took cortical tissue without any fractionation and simply looked at whole tissue lysate by western (again using the 22C11 APP antibody). This revealed the expected increase in levels of APP-FL in the APP/PS1 mice with no reduction in APP-FL expression when comparing APP/PS1 vs. APP/PS1xGFAP-Nrf2 mice (Fig. 7I). As a further control we studied RNA-seq reads spanning the APP KM/NL Swedish locus across all four genotypes under study, calculating the proportion that contained wild-type codons (from endogenous mouse APP) and mutant codons (from the mutant APP transgene). In the APP/PS1 mouse approximately 50% of all APP reads contained the mutation (with negligible numbers in the WT as expected), and this proportion was also approximately 50% in the APP/PS1_X_GFAP-Nrf2 mouse (Fig. 7J). Thus, the protective effects of astrocytic Nrf2 expression with regard to plaque burden are unlikely to be attributable to repression APP expression, raising the question as to what the mechanism may be.

**Fig. 8:**
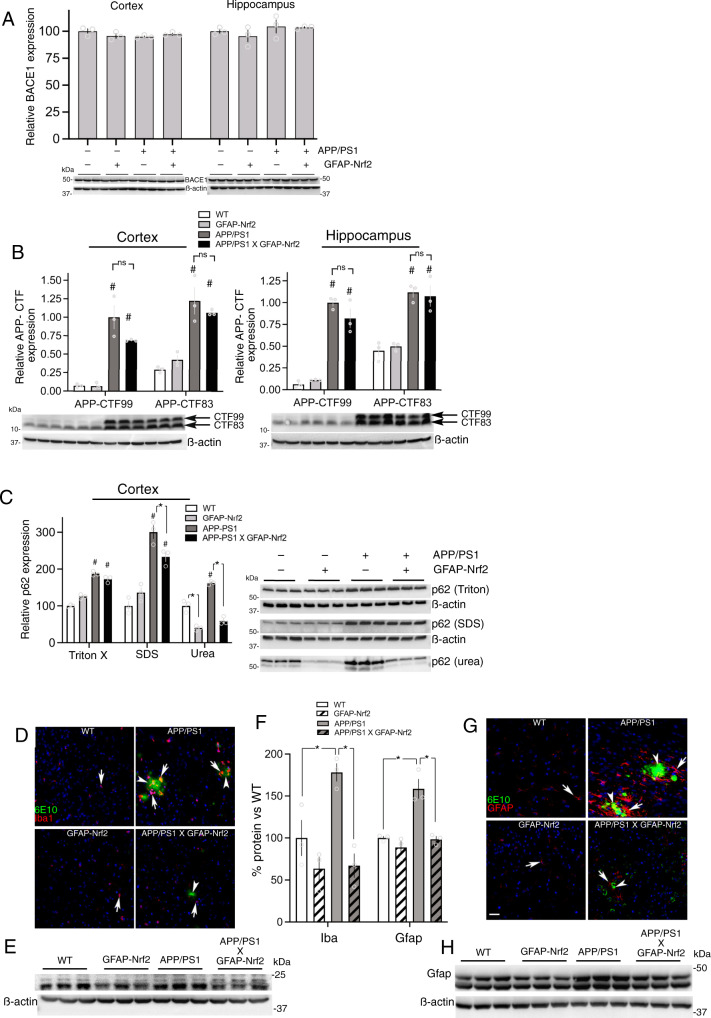
Astrocytic Nrf2 does not alter APP processing but boosts autophagy. **A** Western analysis of BACE1 expression. *F* (1,8) = 0.99, *p* = 0.35 (APP/PS1 effect-cortex); *F* (1,8)=0.38, *p* = 0.55 (GFAP-Nrf2 effect-cortex); *F* (1,8) = 2.24, *p* = 0.17 (APP/PS1 effect-hippocampus); *F* (1,8) = 0.43, *p* = 0.53 (GFAP-Nrf2 effect- hippocampus); Mean ± SEM shown here and throughout this figure**. B** Western analysis of 99 and 83 amino acid C-terminal fragments of APP (antibody C1/6.1) in the cortex (left) and hippocampus (right). Cortex: two-way ANOVA *F* (3, 16) = 53.98, *p* = 12.4E−08 (main genotype effect). ^#^*p* = 2.1E−07, 3.6E−06, 1.7E−05, 3.8E−05 (Bonferroni’s post-hoc test, *n* = 3); ns p values: 0.14, >0.99. Hippocampus: two-way ANOVA *F* (3, 16) = 77.88, *p* = 9.2E−10 (main genotype effect). ^#^*p* = 9.2E−06, 9.8E−04, 8.6E−06, 8.3E−05 (vs. WT, Bonferroni’s post-hoc test, *n* = 3); ns *p* values: 0.46, >0.99. **C** Analysis of p62 in biochemical fractions. For each fraction a two-way ANOVA was performed, showing a main effect of the APP/PS1 genotype: (Triton: *F* (1, 8) = 346.3, *p* = 9.8E−07; SDS: *F* (1, 8) =109.4, *p* = 3.1E−08; urea: *F* (1, 8) = 45.1, p = 0.0002), and interaction of GFAP-Nrf2 and APP/PS1 status (Triton: *F* (1, 8) = 31.5, *p* = 0.0005; SDS: *F* (1, 8) =13.2, *p* = 0.0066; urea: *F* (1, 8) = 12.55, *p* = 0.0076). ^#^*p* = 2.7E−07, 3.2E−05, 1^.^7E−05, 0.0026, 0.0002 (effect of APP/PS1, Bonferroni’s post-hoc test). *p = 0.020, 0.0002, 4.2E−06 (effect of GFAP-Nrf2, Bonferroni’s post-hoc test). *N* = 3 animals/genotype. **D** Immunofluorescence staining of cortical sections for Iba1 (red) and amyloid (green, 6E10 antibody). Arrows indicate Iba1 immunofluorescent cells; arrowheads show 6E10 staining in plaques and neurons. Scale bar: 10 μm. **E***,* **F** Western analysis of Iba1 expression. Two-way ANOVA for Iba1: *F* (1, 8) = 7.1, *p* = 0.029 (APP/PS1 effect); *F* (1, 8) = 23.2, *p* = 0.0013 (GFAP-Nrf2 effect). *p values: 0.014, 0.0018 (Bonferroni’s post-hoc test, *n* = 3). **G** Immunofluorescence staining of cortical sections for Gfap (red) and amyloid (green, 6E10 antibody). Arrows indicate Gfap-positive cells; arrowheads show 6E10 staining in plaques and neurons. Scale bar: 10 μm. **H** Western analysis of Gfap expression; quantitation in Fig. 8F. Two-way ANOVA for Gfap: *F* (1, 8) = 23.5, *p* = 0.0013 (APP/PS1 effect); *F* (1, 8) = 25.7, *p* = 0.0010 (GFAP-Nrf2 effect). *p values: 0.014, 0.0006 (Bonferroni’s post-hoc test, *n* = 3).

As noted above, levels of both Aß40 and Aß42 are lower in all fractions in the APP/PS1_X_GFAP-Nrf2 mouse (Fig. 7G, H), which could be due to altered processing or increased clearance. To investigate the possibility of altered processing of APP into amyloidogenic vs non-amyloidogenic forms, we first looked at BACE1 levels, responsible for the amyloidogenic processing of APP and found no difference in expression in APP/PS1 vs. APP/PS1xGFAP-Nrf2 mice (nor indeed in WT or GFAP-Nrf2 mice, Fig. 8A). BACE1 is involved in processing APP-FL to the 99 amino acid C-terminal fragment of APP (APP-CTF99) which is then processed to Aß, whereas the non-amyloidogenic pathway involves the processing of APP-FL to a 83 amino acid C-terminal fragment (APP-CTF83) by α-secretase. We measured APP-CTF99 and APP-CTF83 levels and did not find evidence for differences between APP/PS1 and APP/PS1xGFAP-Nrf2 with regard to APP-CTF99 or APP-CTF83 levels (Fig. 8B), suggesting that APP processing is not being altered by astrocytic Nrf2, despite lower levels of Aß accumulation at later disease stages (Fig. 7). We then studied levels of Aß at an early disease stage (4 months) before significant plaque deposition, reasoning that if Aß is indeed not being altered, then this is more likely to be apparent before levels rise to levels that invoke clearance mechanisms. As expected, Aß42 levels were far lower at this early stage, and we observed no reduction in Aß42 in either cortex or hippocampus in Triton or SDS-soluble fractions comparing APP/PS1 and APP/PS1xGFAP-Nrf2 mice (Supplementary Fig. 8A), and a modest reduction in the level of Aß42 in the urea fraction of the cortex. Similar results were seen with Aß40 (Supplementary Fig. 8B). Thus crossing GFAP-Nrf2 onto the APP/PS1 line does not appear to influence full-length APP protein expression, nor BACE1 expression or generation of the BACE-mediated pre-amyloidogenic precursor APP-CTF99, and in early disease Aß levels are minimally affected. Collectively this points to the possibility that enhanced degradation or clearance is a contributor to the reduced pathology seen in later-stage disease.

Macroautophagy is an important mechanism for APP and Aß protein clearance, and deficits in autophagy have been reported in AD and AD models (potentially due to improper lysosomal fusion or acidification) leading to a failure of autophagosome degradation. Moreover, manipulating autophagy has been proposed as a potential therapy for amyloidopathies^42–45^. It is of interest that within the subset of Aß/Tau-induced astrocytic genes that were also induced in GFAP-Nrf2 astrocytes, Protease, Lysosome and Autophagy pathways are enriched, (Supplementary Fig. 4), particularly as Nrf2 is a known regulator of autophagy^25^. Protein p62 (gene name: *Sqstm1*) is an autophagy cargo protein that becomes degraded during successful autophagic clearance, so we studied its location on our biochemical fractions. In the APP/PS1 cortex, p62 accumulates in the SDS-soluble and urea-soluble protein fractions, similar to Aß, indicative of unsuccessful autophagy (Fig. 8C). Of note, in the APP/PS1xGFAP-Nrf2 mouse, free p62 levels (Triton-soluble fraction) are similar to those in APP/PS1, however p62 accumulation in the SDS-soluble fraction is reduced, and nearly eliminated in the urea-soluble fraction (Fig. 8C), suggesting that driving astrocytic Nrf2 may promote successful autophagy and prevent accumulation of autophagosomes into SDS-insoluble inclusions. Double-labelling using antibodies against GFAP and the Aß40/42 (using the 4G8 antibody) did reveal 4G8 positive staining in GFAP-positive astrocytes in both APP/PS1 and APP/PS1xGFAP-Nrf2 (Supplementary Fig. 8C), evidence of the capacity for uptake in agreement with others^46^. However, since a difference in presence of 4G8 staining could either mean increased uptake, or failure to execute degradation, it is hard to make conclusions other than that uptake is detectable in astrocytes and that its control by Nrf2 requires further investigation.

Aß pathology is known to trigger chronic neuroinflammation through several potential mechanisms, including the activation of microglia^47^. Additionally, both activated microglia and Aß directly can feedback and influence astrocyte reactivity^2,47^. Thus, we studied the presence of microglial activation/proliferation (Iba1 staining) and sclerotic/reactive astrocytes (GFAP protein positive) in APP/PS1 mice and the effect of astrocytic Nrf2 overexpression on these measures. Iba1-positive microglia were increased in the APP/PS1 mouse, particularly (but not exclusively) in the vicinity of amyloid plaques (Fig. 8D), and increased Iba1 protein was confirmed by western blot (Fig. 8E, F). Moreover, Iba staining and expression was reduced in the APP/PS1_X_GFAP-Nrf2 mouse (Fig. 8D–F), indicative of reduced inflammation. There was a similar pattern in terms of Gfap expression: i.e. an increase in the APP/PS1 mouse revealed by immunostaining and western blot, that was reduced by astrocytic Nrf2 expression (Fig. 8F–H). Of note, Gfap protein expression in the GFAP-Nrf2 mouse was no different to wild-type, despite its transcriptome being significantly enriched in reactive astrocyte genes (Fig. 4C right). The reason for this is discussed further below, but could be due to Nrf2 preferentially driving the adaptive-protective aspects of reactive astrocyte genes without driving Gfap protein expression. We also observed an amelioration of neuronal deficits, with the reduction of neurofilament NF200, and synaptic markers Synapsin-1 and PSD-95 seen in the APP/PS1 cortex rescued in the APP/PS1_X_GFAP-Nrf2 mouse (Supplementary Fig. 8D).

**Fig. 9:**
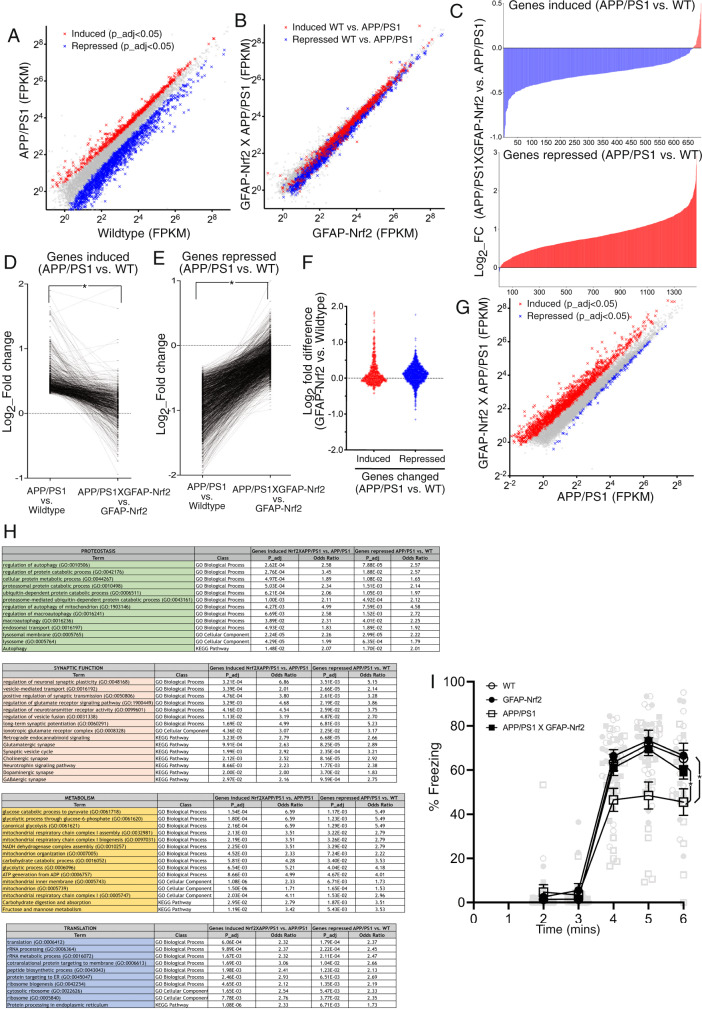
Astrocytic Nrf2 reduces Aß-induced transcriptional perturbation and cognitive deficits. **A** RNA-seq analysis of the hippocampus (APP/PS1 vs WT). Genes induced (red) and repressed (blue) are highlighted (p_adj<0.05; *n* = 4). **B** RNA-seq analysis of early-stage hippocampus (APP/PS1_X_GFAP-Nrf2 vs. GFAP-Nrf2). Genes induced (red) and repressed (blue) from **A**  are highlighted; *n* = 4. **C** For genes in **A**  induced (upper) and repressed (lower), the effect of GFAP-Nrf2 is shown (i.e. APP/PS1_X_GFAP-Nrf2 vs. APP/PS1). *t* = 38.57, df = 682, *<1E−15 (upper); t = 87.95, df = 1455, *p < 1E−15 (lower), ratio paired *t* test (*n* = 4). Log2-fold change (APP/PS1 vs. WT) compared to log2 fold change (APP/PS1 vs. APP/PS1_X_GFAP-Nrf2) for genes induced (**D**) or repressed (**E**) (see Fig. 9A). *t* = 28.51, df = 993, *p < 1E−15 (**D**); *t* = 77.08, df = 1464, *p < 1E−15. **F** Difference in expression (GFAP-Nrf2 vs. WT) of genes induced (red) or repressed (blue) in the APP/PS1 mouse (i.e. genes from **A**). **G** RNA-seq analysis of the hippocampus (APP/PS1 vs APP/PS1_X_GFAP-Nrf2, *n* = 4). **H** GO and KEGG pathways present (p_adj < 0.05) in enrichment analysis of genes downregulated in astrocytes by Aß (APP/PS1 vs. WT, **A**) that were also upregulated by astrocytic Nrf2 in the APP/PS1 mouse (GFAP-Nrf2_X_APP/PS1 vs. APP/PS1, **G**), with pathways grouped under themes (Supplementary Data 12). **I** Fear conditioning test (see Methods). Data are presented as percent freezing during cue testing in 1 min bins starting 60 s after being placed in the chamber (mean ± SEM): WT(*n* = 18); GFAP-Nrf2 (*n* = 13); APP/PS1 (*n* = 13); APP/PS1_X_GFAP-Nrf2 (*n* = 11) mice. Average % freezing across bins 4–6 was calculated for a one-way ANOVA (main genotype effect): (*F* (3, 51) = 6.06, *p* = 0.0013). *P* values: WT vs GFAP-Nrf2, *p* > 0.99; GFAP-Nrf2 vs. APP/PS1_X_GFAP-Nrf2, *p* > 0.99; WT vs APP/PS1, *p = 0.0022; APP/PS1 vs APP/PS1_X_GFAP-Nrf2, *p = 0.040 (Bonferroni’s post-hoc test).

Having studied the impact of astrocytic Nrf2 on APP/PS1 pathology we next investigated the changes at the transcriptional level. We identified genes up- and downregulated globally in the hippocampus (Fig. 9A, B) and cortex (Supplementary Fig. 9A, B) of the APP/PS1 mouse (by bulk RNA-Seq) in an otherwise wild-type background (WT vs. APP/PS1, Supplementary Data 9 and 10). As with the tauopathy model, we then then assessed the perturbation of these genes in a GFAP-Nrf2 background in the hippocampus and cortex (GFAP-Nrf2 vs. APP/PS1_X_GFAP-Nrf2, Fig. 9B, Supplementary Fig. 9B), and found that astrocytic Nrf2 overexpression substantially rescued this global transcriptomic perturbation driven by Aß pathology. As with the MAPT^P301S^ model, clear separation of induced (red) and repressed (blue) genes in Fig. 9A and Supplementary Fig. 9A is largely absent in Fig. 9B and Supplementary Fig. 9B, respectively. Again, the rescue is illustrated by the fact that induced genes are largely repressed by astrocyte Nrf2 expression (Fig. 9C upper, Supplementary Fig. 9C, upper) and repressed genes are largely enhanced by astrocyte Nrf2 expression (Fig. 9C lower, Supplementary Fig. 9C, lower). Consistent with this analysis, fold-induction of APP/PS1-induced genes and fold-repression of APP/PS1-repressed genes is lower in mice overexpressing Nrf2 in astrocytes in both hippocampus (Fig. 9D, E) and cortex (Supplementary Fig. 9D, E). Basal expression of APP/PS1-induced and APP/PS1-repressed genes is largely unaffected in GFAP-Nrf2 mice (Fig. 9F Supplementary Fig. 9F), ruling out changes in the basal denominator as a cause of difference in fold change. Thus, astrocytic Nrf2 rescues global transcriptional perturbation in APP/PS1 mice, as it does in MAPT^P301S^ mice. Also, like the MAPT^P301S^ brain, we did not observe a reduction in reduced glutathione levels in APP/PS1 cortex (Supplementary Fig. 9G), suggestive of a protective effect of astrocytic Nrf2 more complex than rectifying redox imbalance.

To define what gene categories perturbed by Aß pathology were rescued by astrocytic Nrf2, we identified GO and KEGG pathways present (p_adj < 0.05) in enrichment analysis of genes downregulated by Aß pathology (APP/PS1 vs. WT, p_adj<0.05, Fig. 9A) that were also present in analysis of genes upregulated by astrocytic Nrf2 in the APP/PS1 mouse (APP/PS1_X_GFAP-Nrf2 vs. APP/PS1, Fig. 9G, Supplementary Data 11). Collectively, these analyses suggest that Aß pathology induces tissue-wide deficits in four primary areas that are all rescued by astrocytic Nrf2 (Fig. 9H, Supplementary Data 12): protein degradation (proteasome, autophagy, endosomal/lysosomal), carbohydrate metabolism (oxidative phosphorylation, glycolysis, pentose-phosphate pathway), protein translation (including ribogenesis), and synaptic function (pre- and post-synaptic, plasticity, glutamatergic, GABAergic, dopaminergic and cholinergic synapses). Rescue of protein degradation capacity by astrocytic Nrf2 is consistent with the reduced Aß and Tau pathology observed (Figs. 5 and 7). Deficits in energy metabolism, protein synthesis, and synapse function are known facets of AD and AD models, and implicated in causing cognitive decline^48–51^, so their rescue by astrocytic Nrf2 would be biologically significant.

Finally, we wanted to determine if cognitive deficits in the APP/PS1 could be rescued by astrocytic Nrf2. We employed a fear conditioning memory test in which we observed a deficit at 9 months (a 7 month cohort showed a trend towards a deficit that was not significant, Supplementary Fig. 9H). After the training protocol, context and cue testing were evaluated in the four genotypes. For context testing, individual mice were placed back into the same chamber for 5 min but no stimuli were presented. The average percent freezing across the five 1-minute bins ranged from 12–15% and with no significant differences between the groups (Supplementary Fig. 9I). For cue testing, the chamber was changed in several ways prior to testing. Mice were placed in the newly configured chamber. Three minutes after placement in the chamber, the white noise conditioned stimulus was presented for 180 s, but the unconditioned stimulus was not presented. The 9-month-old APP/PS1 mice showed significantly impaired fear learning and memory and this was reversed in the APP/PS1_X_GFAP-Nrf2 mice back to wild-type levels (Fig. 9I). Collectively these data show that Nrf2-driven reactive astrocytes can alter the trajectory of both models of proteopathy at the level of whole-animal behaviour, consistent with the observed effect in slowing patho-progression.

## Discussion

Reactive astrocytes can suppress or exacerbate neuropathology, although what determines this is not well understood^52^. Over the years it has become clear that classical markers such as Gfap immunoreactivity are helpful at defining the extent of astrocytic reactivity, but not at describing the diverse array of responses and phenotypic changes that have occurred, depending on the nature of the insult, the disease time-course or the response of other brain cell types in the immediate environment^9^. Here, the profiling of astrocyte responses to both Tau and Aß pathology revealed that their ‘reactive’ status contains signatures associated with cellular dysfunction and inflammation, and also adaptive-protective responses, the latter of which, if harnessed, can slow both types of pathology central to AD.

### Neither Tau nor Aß pathology induce astrocytes to adopt a profile that fits the A1/A2 hypothesis

Faced with a diversity of cellular phenotypes, it is potentially helpful to categorise them. Recently two types of reactive astrocytes were described: a type that is neurotoxic and defined as the reactivity induced acutely by LPS-activated inflammatory microglia (so-called A1)^19^, and a type which is induced acutely by ischaemia, and proposed to be neuroprotective (so-called A2), albeit with limited evidence^53^. These reactive types display transcriptional signatures that have similarities (pan-reactive genes) as well as genes induced more strongly in either stimuli, with 12 or 13 of each category reported^19^, derived from an earlier microarray study^20^. Chronic neurodegeneration involves reactive astrogliosis that is proposed to be dominated by the LPS-induced signature, although this was based on the presence of putative marker gene C3 in end-stage disease^19,20^. We initially looked at the genes chosen to represent each ‘type’ of astrocyte signature^19^, of which some were discarded due to low expression (<1FPKM) within our astrocyte TRAP-seq data. Of these marker genes, 4/12 and 8/11 (LPS/A1), 6/8 and 1/5 (MCAO/A2), and 9/12 and 7/10 (pan-reactive) were upregulated in MAPT^P301S^ and APP/PS1 astrocytes, respectively. However, not only are these gene sets small (limiting the power for enrichment analyses), the rationale behind their choice was not clear^19^. We therefore exploited the full transcriptional characterisation of these two reactive astrocyte phenotypes^20^ to define larger gene sets. Our expanded analyses showed significant enrichment in all gene sets curated, with no evidence of preferential enrichment in the acute-LPS gene set. This argues against a binary classification of reactive astrocytes into LPS-like (“A1”) or stroke-like (“A2”) as being relevant to chronic disease, and the concept may be an over-simplification in describing astrocytic changes in neurodegenerative disease, in agreement with recent studies and analyses^54,55^.

### Relating astrocyte responses to Tau and Aß pathology to ageing and human AD

Astrocytes from both MAPT^P301S^ and APP/PS1 mice displayed evidence of an induction of genes normally only elevated in astrocytes from very old mice. The classical view of AD as a form of accelerated ageing does not stand up to close scrutiny due to clear differences between AD and ageing with regard to cognitive decline, grey and white matter changes, as well as alterations to functional connectivity and neurotransmission^56^. However, the fact that the molecular response of astrocytes to Tau and Aß pathology overlaps with their response to ageing is consistent with the ageing brain being an environment compromised in some ways (be it metabolic, inflammatory or other homoeostatic functions), as well as with age being the primary risk factor for AD.

Comparison of astrocytes from MAPT^P301S^ and APP/PS1 mice with the largest reported gene set to-date of differentially induced genes in human astrocytes from post-mortem (PM) brains^7^ revealed that both types of pathology induce changes enriched in PM astrocytes (Fig. 3C). Of note, we observed that significant numbers of genes induced in PM AD astrocytes were induced in only one of the models, with a roughly even number specific to either MAPT^P301S^ or APP/PS1 mice (Fig. 3C, right), consistent with human AD being a combination of Aß and Tau pathology. However, it is important to note that human cellular responses to Aß and Tau are likely to exhibit some differences to those seen in mouse models (e.g. due to evolutionary divergence in gene promoter sequences^57,58^), something recently shown in the context of microglia from human AD when compared to those in the 5XFAD mouse model^59^. Such differences may be functionally relevant to AD and require further investigation. Also, since tau and Aß cooperate in animal models of AD^51,60^, further studies will be needed to determine if astrocytes respond to combined tau and Aß pathology in ways that are not simply the sum of their responses to each pathology individually. Nevertheless, there is evidence that activation of Nrf2 in astrocytes, a key focus of our study, occurs in human AD. Expression of Nrf2 target gene NQO1 was observed to be elevated in astrocytes of the frontal cortex of AD patients relative to control, but not in brain areas not affected by AD (e.g. cerebellum)^61^. Moreover, expression of Nrf2 target gene HMOX1 was observed to be elevated in astrocytes of the temporal cortex and hippocampus of both patients with AD and mild cognitive impairment (MCI)^62^. In particular the elevation in MCI patients suggests that astrocytic Nrf2 activation may occur relatively early in disease trajectory, rather than a downstream consequence of end-stage AD.

One interesting distinction between the astrocyte translatome in tauopathy vs. ß-amyloidopathy, was enrichment in AD risk genes and sub-threshold risk loci in the latter, but not the former. This distinction was also made in the context of microglia in a separate recent study^22^. The fact that AD risk genes and sub-threshold risk genes are induced in response to Aß pathology in both microglia and astrocytes in the absence of significant neurodegeneration points to them being involved in a relatively early AD-relevant pathway. It remains to be seen how individual SNPs influence responses of genes to Aß, and what the functional consequences of this are for the astrocytes. However, as noted in the case of microglia^22^, it is possible that cumulative genetic risk determines the response of astrocytes to Aß (direct or indirect e.g. via microglia), thus influencing the downstream cascade that can lead either to a spectrum of outcomes ranging from aggressive AD to apparent tolerance of a high Aß burden in the absence of cognitive symptoms^63^.

### Astrocyte responses to Tau and Aß pathology include a common multi-faceted signature

Substantial numbers of genes were identified in the astrocyte translatome of MAPT^P301S^ and APP/PS1 astrocytes that were induced by both types of pathology, and this set is also enriched (around 30-fold) in a recently curated ‘chronic neurodegeneration’ signature derived from a meta-analysis of astrocyte transcriptomic data from ALS, Tau and Aß models^54^. Gene ontology analysis pointed to activation of inflammatory pathways but also induction of genes associated with protein degradation, causes of which are likely to be multi-faceted. For example, inflammatory pathway activation may be induced in astrocytes indirectly via activated microglia, while protein degradation pathways could be an adaptive/protective response to increased protein aggregates and/or neuritic dystrophies in the extracellular milieu. Microglial involvement in astrocytic transformation requires further investigation, and the recently described mouse (with a Csfr1 promoter element deletion) lacking microglia offers a useful tool in this respect^64^, for example if crossed onto slowly progressing models of Aß pathology.

In the context of genes downregulated by both pathologies, gene ontology analysis pointed to the repression of fundamental processes of protein translation and mitochondrial oxidative phosphorylation. The upstream causes of these changes are a matter of speculation. It is known that glucose metabolism in the brain is lower in AD patients^48–50^, so downregulation of translation machinery and oxidative phosphorylation capacity could reflect cells adapting to a bioenergetically compromised brain environment. What might the downstream consequences of defective astrocytic translation or mitochondrial oxidative phosphorylation in astrocytes be? Impaired translation capacity may impact on the function of astrocytes to provide homoeostatic support of neurons. With regard to oxidative phosphorylation, use of a glia-specific mitochondrial toxin to impair oxidative phosphorylation in glia was reported to cause inhibition of synaptic transmission through the release of adenosine^65^. It has been postulated that this represents an energy-conserving neuroprotective response to prevent lethal depolarisation, though one can envisage that it would nevertheless alter circuit function and cognitive performance if maintained over a long period of time.

Another area of ongoing interest for the field is in CSF biomarkers for AD, which have classically centred on phospho-tau and Aß40/42. However, several genes induced in astrocytes by tau and/or Aß pathology have been reported to have utility as potential fluid biomarkers for AD, Chil1’s 1:1 orthologue CHI3L1 (also known as YKL-40), and Icam1 are both induced in the MAPT^301S^ mouse and are putative CSF/blood AD biomarkers^66^. Of genes induced in both Aß and tau models, Gfap is elevated in the CSF of AD and FTD patients^67^ and C1q and C4b are elevated in plasma astrocyte-derived exosomes in patients with AD^68^ and in patients transitioning from MCI to AD, compared to those with stable MCI^69^. CSF proteins reflecting the trajectory of astrocytic changes in AD and other neurodegenerative disorders may contribute to a panel of validated biomarkers which chart the changes that happen in multiple cell types of the brain, and point to the efficacy of therapies (including combination therapies) on different aspects of pathology.

### Potential mechanisms of disease modification by astrocytic Nrf2

In silico analysis of genes induced in astrocytes by both Tau and Aß pathology pointed to involvement of Nrf2 in regulation of a subset of these genes. Moreover, gene expression changes observed in astrocytes isolated from the GFAP-Nrf2 mouse were enriched in genes induced by both Tau and Aß pathology, as well as in genes induced in human PM AD astrocytes, pointing to Nrf2 activation being evident in response to AD-relevant pathology, and consistent with prior studies showing astrocytic Nrf2 target gene induction in AD (and MCI) patients^61,62^. Nrf2 can be activated by multiple stressors relevant to AD and AD models, including reactive oxygen species and heavy metal dyshomeostasis, acting on Nrf2’s inhibitor Keap1^27,70^. These stressors can in turn be triggered by several processes in AD including bioenergetic dysfunction, inflammation or vascular impairment. Nrf2 drives the expression of a large number of genes associated with antioxidant production, xenobiotic detoxification, metabolic reprogramming, and proteostasis, and its activation is able to exert both cell-autonomous and non-cell-autonomous cytoprotection^25–27,71^. However, the presence of Nrf2 target gene signatures in astrocytes in human AD and AD models is suggestive of a response that is too little, too late, to substantially alter disease trajectory. In contrast, we show here that pre-emptive activation of Nrf2 in astrocytes is sufficient to alter disease trajectory in models of both ß-amyloidopathy and tauopathy, raising the question as to the mechanism(s) involved. One potential contributing factor is a cell-autonomous increase in uptake and degradation of toxic protein moieties via the lysosomal system. At the gene expression level this pathway forms part of the signature in astrocytes associated with Nrf2 targets genes, and so Aß clearance may be enhanced in astrocytes via this mechanism. However, there are other pathways that may be influenced in a non-cell-autonomous manner to alter pathology and progression of ß-amyloidopathy. Astrocytic Nrf2 activation promotes glutathione production and release into the extracellular space, which has the dual effect of boosting extracellular antioxidant capacity, as well as providing cysteine-based precursors for neurons and other cells to use to enhance their own glutathione production^72^ (capacity of which is regulated by other mechanisms such as synaptic activity^73,74^). In this way, both interstitial redox buffering can be enhanced as well as the antioxidant capacity of non-astrocytic cells. Increased redox buffering and enhanced antioxidant defences in the extracellular space can reduce Aß aggregation^75^. Moreover, increased intracellular redox buffering can repress cytotoxicity and unfolded protein response (UPR) over-activation in response to ER stress inducers, at least in part through maintenance of ER redox balance and disulphide chemistry^76–78^, as well as control autophagic clearance of misfolded proteins and activity of the ubiquitin proteasome^79^. Many of these pathways are also relevant to mutant Tau aggregate clearance: redox state is known to influence both tau phosphorylation and aggregation^80^. Moreover, interactions of Nrf2-overexpressing astrocytes with other cells in the brain can ultimately influence patho-progression. It is worth noting that we did not observe differences in brain glutathione levels in either model of Tau or Aß pathology, raising the possibility that astrocytic Nrf2’s influence on the redox environment is local and/or subtle, or that it is acting via other mechanisms that it promotes, such as proteostasis. Indeed astrocytic Nrf2 ameliorates pathoprogression in the SOD1^G93A^ model of ALS and hSyn^A53T^ model of Parkinson’s disease^28,81^, raising the possibility that similar protective mechanisms may be responsible across a range of misfolded protein disorders. That said, future studies on the influence of Nrf2 on disease trajectory need to address the issue of whether activation later in the disease stage can also be protective.

To conclude, the ability of astrocytes to mitigate brain dysfunction by limiting extra-neuronal Aß pathology and intra-neuronal Tau pathology point to their potential to widely influence brain health in the context of AD. The therapeutic targeting of astrocytes offers the potential to influence these key mediators of brain homoeostasis which, potentially in combination with anti-aggregate immunotherapy, or other manipulators of microglial or vascular cell phenotype, may provide an environment that resists the progression of AD, preserving neuronal circuit integrity and cognitive function.

## Methods

### Animal lines

All procedures described were performed either in the University of Edinburgh in compliance with the UK Animals (Scientific Procedures) Act 1986 and University of Edinburgh regulations, and carried out under project license numbers 70/9008 and P1351480E, or in the University of Wisconsin-Madison, approved by their Institutional Animal Care and Use Committee. Mice were group-housed in environmentally enriched cages within humidity and temperature controlled rooms, with a 12-h light dark cycle with free access to food and water. Mouse genotypes were determined using real-time PCR with transgene specific probes (Transnetyx, Cordova, TN) unless otherwise stated. The Aldh1l1-EGFP-Rpl10a transgenic mouse line expresses an EGFP-tagged ribosomal protein l10a under the astrocyte-specific aldh1l1 promoter, was generated as previously described by the laboratory of Nathaniel Heintz^17^ and re-derived on a C57B/6J background from frozen sperm imported from Jackson Laboratories (Mouse Strain Number – 030248). Animals were utilised as heterozygotes in all experiments. The Thy1-hTau.P301S transgenic mouse line (C56BL/6J background) over-expresses human mutant P301S tau under the neuron-specific Thy 1.2 promoter and was generated by the laboratory of Michel Goedert^10^. Female mice were used for all experiments as they both demonstrate less fighting and exhibit more rapid-onset pathology than males. All animals used were homozygous at 12 or 20 weeks of age being culled by perfusion with ice-cold PBS with cycloheximide, followed by rapid dissection of the superficial frontal cortices and cervical spinal cord (C2–C7 vertebral levels) for fresh tissue or for fixed tissue mice were perfused initially with PBS prior to a 4% paraformaldehyde solution in PBS being pumped in. Brain and spinal cords were removed and post-fixed overnight in 4% PFA prior to being cryoprotected in 30% sucrose solutions and tissue being frozen down ready for cryosectioning. The GFAP-Nrf2 mouse line GFAP-Nrf2 transgenic mice (C56BL/6J background) were created previously^28^. by insertion of the mouse Nrf2 gene downstream of the 2.2 kb segment of the human GFAP promoter. APP/PS1 mice carrying a chimeric mouse/human APP (Mo/HuAPP695_swe_) and a mutant human presenilin 1 (PS1-_dE9_)^13,82,83^, were purchased from The Jackson Laboratory (B6.Cg-Tg(APPswe,PSEN1dE9)85Dbo/Mmjax; MMRRC Stock No: 34832-JAX). Where Transnetyx were not employed, the following genotyping primers were used: GFAP-Nrf2 (Fw: 5′-CTT CAT AAA GCC CTC GCA TC-3′) and (Rv: 5′-TCT TGC CTC CAA AGG ATG TC-3′) which results in a band at 400 bp in GFAP-Nrf2 positive mice. The primers for APP are (5′-GAC TGA CCA CTC GAC CAG GTT CTG-3′) and APP (5′-CTT GTA AGT TGG ATT CTC ATA TCC-3′) which results in a band at 350 bp in APP transgenic mice. The primers for PS1 are (5′-GCC ATG AGG GCA CTA ATC AT-3′) and (5′-ATT AGA GAA CGG CAG GAG CA-3′) which results in a band at ~ 608 bp in PS1 transgenic mice. Jackson lab internal control Primers are (olMR0015: 5′-CAAATGTTGCTTGTCTGGTG) and (olMR0016: 5′-GTCAGTCGAGTGCACAGTTT) which gives a band at 210 bp in all mice.

### Tissue culture

Astrocytes and neurons were cultured as previously described^84^. In brief, cortices from E17.5 pups were dissected following decapitation in dissociation medium DM (mM: 81.8 Na2SO4, 30 K2SO4, 5.84 MgCL2, 0.252 CaCl2, 1 HEPES, 0.001% Phenol Red, 20 Glucose, 1 kyurenic acid, adjusted to pH 7.35 with NaOH). Tissue was enzymically digested by incubation with DM with 10 U/mL papain enzyme (Worthington Biochemicals) and plated on poly-D-Lysine and Laminin (Sigma) coated plates. Neuron cultures were maintained in Neurobasal-A medium containing B-27 (Life Technologies), 1% Rat Serum, and treated with 4.8 µM of the anti-mitotic cytosine-arabinoside (AraC) to limit glial proliferation and ensure >99.8% NeuN-positive neurons. Astrocytic cultures were maintained in DMEM with 10% Foetal Bovine Serum (Life Technologies) for 7 days, dissociated with Trypsin and sub-cultured twice to obtain > 98% pure GFAP-positive astrocytes.

### Translating-ribosome affinity purification

Isolation of cell-type-specific translating mRNA was carried out as previously described^85^. Briefly: tissue or cells were lysed in ice-cold lysis buffer (in mM: 20 HEPES, 10 MgCl_2_, 150 KCl, 0.5 DTT) along with 100 μg/mL cycloheximide (Sigma), cOmplete ULTRA protease inhibitors (Sigma) and RNAse inhibitors (Superasin – Life Technologies; RNAsin – Promega). Cell lysate was centrifuged to clear debris and solubilised with 1% NP40 and 30 mM 1,2-diheptanoyl-*sn*-glycero-3-phosphocholine (DHPC—Avanti Polar Lipids). Following further centrifugation, supernatants were added to pre-prepared anti-GFP (19C8 and 19F7 antibodies – Sloan Kettering Memorial Centre) coated magnetic beads (Dynabeads MyOne Streptavadin T1—Life Technologies). Following immunoprecipitation overnight at 4 °C with continuous rotation, beads were washed four times. Cell-type-specific mRNA, attached to immunoprecipitated GFP-tagged ribosomes, was isolated and purified using the Agilent Nanoprep kit described above. 50 μL of solubilised lysate (“input sample”—representing total RNA) was taken prior to immunoprecipitation and spun overnight at 4 °C (same conditions as TRAP samples) before RNA-purification with TRAP samples.

### RNA extraction

RNA extraction was carried out using the RNeasy Lipid Tissue kit (Qiagen) as per the manufacturer’s instructions. In brief, samples were homogenised in 1 mL of QIAzol Lysis re-agent. Following addition of 200 μL chloroform, and separation by centrifugation, the aqueous phase is collected and an equal part of ethanol added. RNA is extracted and purified by binding and washing through an RNeasy Mini-spin column. The final purified RNA was eluted in 30 μL RNAse free water. Low-yield RNA extraction (for example following ribosomal pulldown) was carried out using the Absolutely RNA Nanoprep kit (Agilent) as per manufacturer’s instructions. Samples were lysed in 100 μL of lysis buffer with β-mercaptoethanol; mixed with equal volume of 80% sulfolane and added to silica-based fibre filter columns. DNAse treatment was carried out for 15 min; columns were washed and dried, and RNA eluted in 20 μL of pre-warmed 60 °C RNAse free water.

### Quantitative RT-PCR

cDNA was generated using the Transcriptor First Strand cDNA Synthesis Kit (Roche). Seven microlitres of RNA was added to the RT and buffer mixture prepared with random hexamers and oligoDT primers as per kit instructions, and rtPCR carried out with the following programme: 10 min at 25 °C, 30 min at 55 °C and 5 min at 85 °C. qPCRs were performed on a Mx3000P QPCR machine (Agilent Technologies) using the FastStart Universal SYBR Green QPCR Master (Rox) (Roche) reagent. Six nanograms of cDNA was used for each reaction and all qPCR results were carried out in duplicate or triplicate, along with no template controls and no RT controls where appropriate. The following cycling programme was used: 10 min at 95 °C; 40 cycles of: 30 s at 95 °C, 40 s at 60 °C (with fluorescence detection), 1 min at 72 °C; ending with dissociation curve: 1 min at 95 °C and 30 s at 55 °C with a ramp up to 30 s at 95 °C with fluorescence detection. All data were normalised to house-keeping gene controls (*Rpl13a*). Primer sequences are detailed below (Table 1):

**Table 1:** Primer sequences.

Gene	Forward (5′–3′)	Reverse (5′–3′)
*Aif1*	GCAATGATGAGGATCTGCC	CCACTGGACACCTCTCTAATTAATC
*Aldh1l1*	GTTGCTAGCCCAGAGCC	GGAACTTAAACACGGGCAC
*Angpt*	AGCAATCCTTAGCATAGGGG	TCTCTGTGTAACCGTTCAGC
*Aqp4*	GAGAGTCGTCACACCAGTG	TCCCAGCCAGGAAGTAACTA
*Cx3cr1*	CTGGTGGTCTTTGCCTTC	GCACTTCCTATACAGGTGTCC
*Dio2*	CCCTTCTGAGCGAATTGATCCA	CACATCGTAAGTATGTATCTGGG
*Eno2*	GCCATCTCCTGTAACTCTCC	ATTCTGTAAAGTTCCGAGCTTC
*Gfap*	GCAAAAGCACCAAAGAAGGGGA	ACATGGTTCAGTCCCTTAGAGG
*Lgals3bp*	CCACATGGTGGAGCTTTTCT	CAACCCTGGGAGCAAGAGA
*Lpl*	TGCCCTACAAAGTGTTCCAT	CAGGGTGAAGGGAATGTTCT
*Mbp*	CCAGTCTAATAATGTCCATCGAC	CAGATTAACAAGATGCAGTATTGG
*Mog*	AAAGAATACCGACCAGAGAAATAC	CACATTGGTTCTCAGAGAAATAAG
*Npy*	AGACCTCTTAATGAAGGAAAGCAC	CAGGCAGACTGGTTTCAGG
*Nrf2*	CAGCTCAAGGGCACAGTGC	GTGGCCCAAGTCTTGCTCC
*Rpl13a*	GATGAATACCAACCCCTCC	CGAACAACCTTGAGAGCAG
*Slc1a2*	TATCATCTCCAGTTTAATCAC	TTCATTCAACATGGAGATGACC
*Slc1a3*	CAAGACACTGACACGCAAGGAC	CTTAACATCTTCCTTGGTGAGGC
*Slc14a1*	CTCGTGACCAAAACCCTTAG	GACCCAACGTTTATTCCACA
*Trim47*	GTTCATCGAAGAGGGAGAGG	CTCCTGGAGAAAGCTGACTG
*Tubb3*	CCGACAACTTTATCTTTGGTC	TCCACCACAGTGTCCG

### RNA-seq and its analysis

Whole-brain RNA sequencing was performed using Trueseq Stranded Total RNA V2 library preparation along with next-generation sequencing on the Illumina Novaseq 6000 platform. At-least 1 μg RNA per sample was utilised, with RNA-integrity number (RIN) > 7. TRAP-sequencing (along with sequencing of matched inputs where appropriate) was performed utilising the Clonetech - SMART-Seq v4 Ultra Low input RNA library preparation, along with sequencing on the Illumina Nextseq 500 platform. At least 1 ng RNA was used per sample with RIN > 7. RNA-seq reads were mapped to genome sequences using STAR (Spliced Transcripts Alignment to a ref. 86). Per-gene read counts were summarised using featureCounts^87^, and differential expression analysis performed using DESeq2^88^, with a significance threshold calculated at a Benjamini–Hochberg-adjusted *P* value of <0.05. Mixed-species sorting was carried out using the SARGASSO (Sargasso Assigns Reads to Genomes According to Species-Specific Origin) python tool as described^38^ *(source code available:* https://github.com/biomedicalinformaticsgroup/Sargasso*)*, which utilises a strategy that aims to minimise reads misallocated to the incorrect species whilst maximising the number of reads unambiguously assigned to the correct species.

### Generation of expanded LPS-, MCAO and pan-reactive gene sets

The original sets of A1 (LPS-specific), A2 (MCAO-specific) and pan-reactive gene sets comprised groups of 12-13 genes^19^ and, although they were derived from the microarray data previously published^20^, the rationale behind their selection was not stated. To generate larger gene sets, the microarray data from GSE accession number GSE35338 previously published^20^ was analysed in GEO2R and fold change gene expression for the acute-LPS and acute-MCAO sets calculated. For genes featuring within the top 250 for either stimuli and for which there were multiple probe sets, the average fold change for the probe sets was taken as the final value. For both stimulation paradigms, fully annotated genes were ranked by fold change (highest fold change ranked highest). To generate LPS and MCAO gene sets, we required the gene be ranked in the top 100 genes for one stimulation paradigm, and ranked at least 50 places lower for the other stimulation paradigm. Using this approach 70 genes were obtained both for the LPS and MCAO sets (Supplementary Data 6). To generate pan-reactive gene sets we wanted robustly induced genes that were of similar ranking across both stimulation paradigms, so required that genes be in the top 250 ranked genes, and no more than 50 ranking positions between the two stimulation paradigms. This yielded a pan-reactive set of 42 genes (Supplementary Data 6). Of the 38 ‘A1’ (LPS), ‘A2’ (MCAO) and pan-reactive genes previously employed^19^, 8 were found in different categories using our method. For example, we classed one previously stated A1/LPS gene (*Gbp*) as a pan-reactive gene since it was ranked 8^th^ (LPS stimulation) and 26th (MCAO stimulation). Conversely, the gene *Serpina3n* was previously classed as a pan-reactive gene, but we classed it as an LPS gene since it was ranked 6th (LPS stimulation) and 61st (MCAO stimulation). Of note, when interrogating TRAP-seq data with these gene sets, only genes within these sets expressed >1FPKM across all conditions were included in the analysis.

### Protein extraction, Aβ fractionation

The tissue was thawed, weighed and processed by sonication in Triton-X-lysis buffer containing 1% Triton^TM^ X-100, 50 mM Tris-HCl, pH 7.6, 150 mM NaCl, 2 mM EDTA, 1 mM DTT, phosphatase inhibitor cocktails 2 and 3 (Sigma) and complete^TM^ Mini EDTA-free Protease Inhibitor Cocktail tablet (Roche) at a ratio of 1:10 (1 mg tissue = 10 μL Triton-X-lysis buffer). The resulting total lysates were assayed for protein concentration using the Pierce^TM^ BCA protein assay. Triton-X-soluble, SDS-soluble and urea-soluble fractions were generated for cortical and hippocampal tissue. Equal amounts of Triton-X-lysate protein (850 µg) in a final volume of 100 µL were centrifuged at 100,000 × *g* for 60 min at 4 °C. The supernatant was collected as the Triton-X-soluble fraction. Pellets were washed with Triton-X-lysis buffer and then re-suspended in equals volumes of Triton-X-lysis buffer containing 1% SDS and centrifuged at 100,000 × *g* for 60 min at 4 °C to generate the SDS-soluble fraction. The remaining pellets were washed with Triton-X-lysis buffer containing 1% SDS and dissolved in buffer containing 30 mM Tris-HCl, pH 8.5, 7 M urea, 2 M thiourea and 4% CHAPS and centrifuged at 100,000 × *g* for 60 min at 4 °C to generate the urea-soluble fraction.

### Enzyme-linked immunosorbent assay

Five microlitres of each fraction (Triton-X, SDS, and urea) was assayed for levels of human Aß40 and Aß42 using ELISA (Invitrogen). Briefly, cortical and hippocampal homogenates were diluted (four volumes) with cold 5 M guanidine-HCL in 50 mM Tris-base (pH 8.0) buffer followed by mixing for 3–4 h on an orbital shaker at room temperature (RT). Per the manufacturer’s instructions, samples were then diluted tenfold with cold PBS containing 1X-protease inhibitor cocktail (Sigma) and centrifuged at 16,000 × g for 20 min at 4 °C. Supernatant was carefully decanted and diluted 1:10 with standard diluent buffer from the ELISA kit and levels of human Aß40 and Aß42 were determined using the manufacturers recommended protocol.

### Luminescence-based reduced glutathione (GSH) quantification

GSH measurements were performed using the GSH-Glo glutathione assay kit (Promega) as per manufacturer’s instructions. In brief, CNS tissue was lysed in Passive Lysis Buffer (Promega). The lysate was centrifuged at 20,000 G for 10 min at 4 °C to remove tissue debris and 50 μL of supernatant added in triplicate to a 96 well plate. Fifty microlitres of pre-prepared 2X GSH-Glo re-agent was added per well, mixed and incubated at room temperature for 30 min. One hundred microlitres of reconstituted Luciferin detection re-agent was added per well and luminescence measured after 15 min using a spectrophotometer and GSH concentrations determined from a standard curve following background subtraction.

### RNAScope

Tissue was serially sectioned and fixed in 10% neutral buffered formalin (ThermoFisher) for a minimum of 24 h, then dehydrated in an ascending alcohol series (70–100%) followed by three successive four-hour washes in xylene. Three successive five-hour paraffin wax embedding stages were performed followed by cooling and sectioning of the formalin-fixed paraffin-embedded tissue on a Leica microtome in 4-μm sections on to a superfrost microscope slide. RNAScope reagents (Advanced Cell Diagnostics) were used as per manufacturer’s guidelines. In brief, following deparafinisation, tissue sections were incubated with hydrogen peroxide for 10 min at room temperature and target antigen retrieval was performed by submerging slides in RNAScope 1× target retrieval reagent at 99 °C in a Braun Multiquick FS 20 steamer for 15 min. The tissue was then permeabilised using RNAScope protease IV at 40 °C for 30 min. Probe hybridisation was then performed by incubating the slides with four drops of custom designed RNAScope probe (to recognise mouse gfap, and hmox1 transcripts) for two hours at 40 °C. Following successive probe amplification steps, transcripts were detected using the RNAScope 2.5 HD duplex detection kit (Chromogenic) and slides were counterstained using haematoxylin (2 min incubation) and lithium carbonate (30 s incubation). The slides were then cleared in xylene and mounted with a 24 × 50 mm coverslip using two drops of VectaMount mounting medium. Sections were then imaged at ×10 and ×40 magnification using a Nikon Eclipse E400 microscope.

### Immunohistochemistry

Primary antibodies are listed in the table below. For MAPT^P^^301S^ studies, secondary antibodies used were Alexa 488, Alexa 555, and streptavidin 555 (1:1000, Invitrogen) in conjunction where appropriate with bisbenzimide (1:5000, Sigma) to identify cell nuclei. All counts and analyses were performed rostral to Bregma in the forebrain of the mice as defined previously^11^. All counts and analyses were performed throughout the cortex and the cortical layers were defined as previously described^11^. Briefly: layers I and II, surface to a depth of 200 µm; layer III, 200–350 m; layer IV, 350–550 m; layer V, 550–750 m; and layer VI, 750– onwards. We refer to the superficial cortex throughout, and this is defined as extending from the surface to a depth of 200 µm, equivalent to layers I and II. At8 counts were performed throughout the whole section in the defined areas as outlined above. Three slices per animal were used (and combined to create a single average). For imaging of Aldh1l1-eGFP-rpl10a slices, animals were killed by perfusion-fixation with 4% PFA. Brains were removed and 50 μM slices prepared using a vibratome. Slices were washed 3x with PBS, blocked and permeabilized with 10% normal goat serum (Thermofisher) with 0.3% Triton-X (Sigma-Aldrich). Primary antibodies were applied overnight at 4 °C whilst shaking, and secondary antibodies applied for 2 h at RT. Following this, cells or slices were washed 4× in PBS and mounted using Vectashield with 4′,6-diamidino-2-phenylindole (DAPI) (Vector Labs). Images were acquired using a Nikon A1R laser scanning confocal microscope. Laser beams of wavelength 488 and 561 nm were used to excite FITC and CY3 fluorophores respectively, using a 20 × 0.8 NA objective lens. NIS elements software was used for the image acquisition and ImageJ software was used for the analysis. For APP/PS1 analysis, coronal sections (10 μm) were prepared using a microtome (Leica). Sections were deparaffinized and rehydrated followed by antigen retrieval in sodium citrate buffer (10 mM sodium citrate, 0.05% TWEEN^®^-20, pH 6.0) at 95–100 °C for 40 min. Alternatively, other sets of samples were incubated for 5 min in 95% of formic acid (Sigma) in PBS. Sections were then permeabilized and blocked with 10% goat serum, 1% bovine serum albumin (BSA), and 0.3% Triton^TM^ X-100 in PBS for 1 h at RT followed by incubation with primary antibodies diluted in the same buffer at 4 °C overnight. Appropriate isotype-specific IgG antibodies were used for negative controls. For light microscopy, sections were processed using the VECTASTAIN^®^ Elite^®^ ABC System as per the manufacturers protocol. Sections were visualised by DAB staining (Vector lab). For fluorescent imaging, the sections were blocked with staining buffer (10% goat serum, 0.4% Triton^TM^ X-100, and 0.5% BSA in PBS) and incubated with the primary antibody in staining buffer at 4 °C overnight. The following day, sections were incubated with appropriate Alexa-Fluor secondary antibodies in staining buffer for 1 h at RT and counterstained with Hoechst 33258 (Invitrogen) prior to coverslipping. Samples were visualised using a Nikon Eclipse T*i*2 fluorescent microscope. For immunohistochemistry in cultures cells, a paraformaldehyde fixation followed by permeabilization (NP40)^89^.

### Aß plaque quantification

Microscopic images of mouse whole-brain sections treated with formic acid and were stained with 6E10 antibody as described above. Sections were visualised and captured by a Nikon Eclipse Ti2 microscope and a DS-Fi3 Brightfield colour camera, respectively. Using an *x*–*y* motorised stage, mouse brain sections were scanned and several hundred images per section were captured at an amplification of 200×. Images were then stitched together using Nikon NIS Elements software, and a single high-resolution panoramic photograph of whole brain sections were then used for quantification of Aß plaque load.

Aß plaque analysis was performed using ImageJ v1.53a (Rasband, W.S., ImageJ, U. S. National Institutes of Health, Bethesda, Maryland, USA, https://imagej.nih.gov/ij/, 1997–2018). Briefly, whole-brain stitched images were thresholded to obtain foreground and background pixelated images (Fig. S7A). Cortex or hippocampus were selected in the thresholded images and Aß plaque sizes of 5–10,000 µm were then measured to obtain total number, percent area, and average size. Three brain slices per mouse were averaged and four mice from each cohort (APP-PS1 and APP-PS1xGFAP-Nrf2) were used for quantification.

### Immunoblotting

For western blot detection of APP and Aβ, samples from Triton-X-soluble fraction; SDS-soluble fraction, and urea-soluble fraction were separated by electrophoresis using a 10% Tris-Tricine polyacrylamide gel. The resolved proteins were transferred to Hybond-P (PVDF) membrane (Amersham) and boiled for 15 min in 1× PBS before blocking. Membranes were blocked for 1 h in Tris-buffered saline, 0.1% TWEEN^®^-20 (TBST), and 5% non-fat dry milk, followed by overnight incubation with primary antibody diluted in the same buffer. Total lysates were resolved using 10% Tris-glycine SDS-PAGE. For ATGs (autophagy-related proteins) and phospho-specific proteins, membranes were blocked with 5% BSA in TBST prior to overnight incubation in primary antibody diluted in the same buffer. After washing with TBST, the membrane was incubated with peroxidase-conjugated secondary antibody (antibody to rabbit and mouse IgG from KPL and goat IgG from SeraCare; Life Sciences Inc.) for 1 h, and then washed and developed using the ECL chemiluminescent detection system, Supersignal^®^ west pico (Thermo Scientific). Blots were visualised using a Syngene G:Box with GeneSys image acquisition software. Densitometric analyses were performed using Adobe Photoshop CC 2018 and normalised against the signal obtained by reprobing the membranes with anti-β-actin. For western blot analysis in experiments involving MAPT^P301S^ samples, total cell lysates were boiled at 100 °C for 5 min in 1.5 × sample buffer (1.5 M Tris pH 6.8; Glycerol 15%; SDS 3%; β-mercaptoethanol 7.5%; bromophenol blue 0.0375%). Gel electrophoresis and western blotting were performed using Xcell Surelock system (Invitrogen) using NuPage Novex 4-12% Bis-Tris gels (Invitrogen) according to the manufacturer’s instructions. The gels were blotted onto PVDF membranes, which were then blocked for 1 h at room temperature with 5% (w/v) non-fat dried milk in TBS with 0.1% Tween 20. The membranes were incubated at 4 °C overnight with primary antibodies diluted in blocking solution and visualised using HRP-based secondary antibodies (1:2000) followed by chemiluminescent detection on Kodak X-Omat film. Western blots were analysed by digitally scanning the blots, followed by densitometric analysis (ImageJ) and total protein normalisation using Memcode Reversible Protein Staining (Thermofisher) as per manufacturer’s instructions. Antibodies, dilutions, techniques, sources, and catalogue/product numbers used are listed below (Table 2):

**Table 2:** Antibodies used.

Antibody	Dilution	Technique	Source	Catalogue/Product #
β-Amyloid 1–16, (6E10)	1:1,000 1:1,000 1:1,000	WB IHC IF	Biolegend	803003
β-Amyloid 17–24 (4G8)	1:500	IF	Biolegend	800708
APP A4 66–81, (22C11)	1:1,000 1:1,000	WB IHC	Millipore	MAB-348
APP 676–695 (C1/6.1)	1:10,000	WB	Biolegend	802801
BACE1	1:1,000	WB	GeneScript	A00879
GFAP (G-A-5)	1:10,000 1:1,000	WB IF	Sigma	G 3892
GFAP GA5-Cy3	1:500	IF	Sigma	MAB3402
Iba1	1:1,000 1:500	WB IF	Wako	019-19741
NQO1	1:1,000	WB	Abcam	ab34173
NQO1 (A180)	1:200	IF	Santa Cruz	sc-32793
GCLM	1:1,000	WB	Dr. Terry Kavanagh	University of Washington
β-Actin (8H10D10)	1:10,000	WB	Cell Signalling Technology	3700
anti-NeuN	1:400	IF	Millipore Bioscience	MAB377
anti-phosphotau AT8	1:1000	IF	Autogen Bioclear	90206
Neurochrom-Cy3	1:500	IF	Millipore Bioscience	ABN2300C3
Anti-Aldh1l1	1:500	IF	Abcam	ab87117
SQSTM1/p62	1:1,000	WB	Abnova	H00008878-B01
NF200 (NE14)		WB	EMD Millipore	N5389
NF200 (N52)	1:1000	WB	EMD Millipore	MAB5266
PSD-95	1:1000	WB	Abcam	ab12093
Synapsin-1	1:1000	WB	Synaptic Systems	106 103

### Fear conditioning

Fear conditioning sessions was conducted in 4 identical commercially constructed chambers (18 cm W × 18 cm D × 28 cm H; Coulbourn Instruments, Whitehall, PA) by the behavioural testing service in the Intellectual and Developmental Disabilities (Liddell) Models Core at the University of Wisconsin Waisman Center. The walls of the chambers are constructed of plexiglass and sheet aluminium, while the floor consist of stainless-steel rods. The floor rods are connected to an adjustable shocker set to deliver 0.7 mA. Mounted on top of each chamber, directed through a circular hole in the aluminium ceiling, are Panasonic digital CCTV cameras (model WV-BP334) interfaced with a PCI-1410 video capture board installed in a Pentium class personal computer. All events were programmed and data recorded through FreezeFrame2 and FreezeView software from Actimetrics Software (Wilmette, IL, USA).

The training protocol used a 6 min-long-2-trial delay conditioning procedure, with co-terminating 30 s white noise conditional stimuli (CS) of 87 dB and 1.5 s shock unconditional stimuli (US) of 0.7 mA; trials were separated by 2 min inter-trial-intervals (ITI). Mice were placed in the chambers and after 2 min, the CS was played for 30 s. Twenty-eight and a half seconds after onset of the CS, a 1.5 s US will be delivered to each floor. This was followed by a 2-min ITI. Then, the white noise CS was again played for 30 s, co-terminating with a 1.5 s US.

For context testing, mice were tested for conditioning to context 20–24 h after training. Context testing lasted 5 min. Individual mice were placed back into the same chamber in which they received the conditioning session, but no stimuli were presented. For cue testing, mice were tested for conditioning to the CS 2 h after context testing. The chamber was changed in several ways. First, a white plexiglass floor panel was placed over the top of the stainless rods. Second, grey-plexiglass panels were inserted into the chamber and affixed to 3 of the four walls (the fourth wall served as the door to the chamber). Third, a small paper towel, with several drops of vanilla extract, was placed below the stainless rod floor in the catch tray. Fourth, the chamber was cleaned with 30% Isopropyl alcohol rather than 70% EtOH. These changes provide a novel environment so that cue testing was minimally affected by conditioning to context. The testing session was 6 min long. Mice were placed in the newly configured chamber. Three minutes after placement in the chamber, the white noise CS was presented for 180 s, but the US was not presented. All sessions were recorded by FreezeFrame2 software by capturing images at a rate of 4 per second (4 Hz).

### Horizontal bar testing

Horizontal bar test was used to assess the limb muscle strength and coordination. Each mouse was gently suspended at the centre of a metal bar by its tail. The mouse was then allowed to grip the metal bar with its forelimb while releasing its tail and allowed to move to one of the platforms on either side of the bar. The bar was 26 cm long, 0.2 cm in diameter and placed at a height of 19.5 cm between two plastic pillars. A metal mesh on the pillars acted as platform. The time taken for the mice to fall from metal bar or time taken to reach the platform from the centre of the bar was recorded and converted into a score between 1 and 10. The scoring system used was a modified and more sensitive version of previously published protocol^90^ so that the time taken to reach the platform was also factored in the score. Scoring criteria for time taken to fall from the bar and the time taken to reach the platform is specified in the table below. The cut-off time for each test was 60 seconds. Each session of testing had 3 tests and the mean score per animal per session was calculated by averaging the score from three tests. The testing session was carried out on a weekly basis for each mouse. For each trial the time taken to reach the platform or time taken to fall from the bar were converted to a score as described in Table 3 and the mean score for that session was calculated for each mouse (three trials per session). This “within animal” mean was used as the primary data in statistical analysis. Piecewise linear regression was used with random intercept for ID to compare the trajectory of the longitudinal performance of the two or four groups.

**Table 3:** Scoring system for horizontal bar testing.

Score	Time to fall (s)	Score	Time to reach platform (s)
0	0–5	10	0–5
1	6–10	9	6–10
2	11–20	8	11–20
3	21–40	7	21–30
4	41–59	6	31–59
5	60	5	60

### Statistics and reproducibility

Throughout the manuscript the statistical test and (if relevant) post-hoc test is stated, along with *p* values, unless below the limit for our statistics software package. All statistical tests performed are two-sided, and performed on biological replicates, not technical replicates. For any post-hoc tests we correct for multiple comparisons (Bonferroni for ANOVAs), and only the comparisons relevant to the hypothesis being tested were performed (rather than comparing all data sets with all others) and the *p* values quoted are always the adjusted *p* value. Differential expression analysis of RNA-seq data was performed using DESeq2^88^, with a significance threshold calculated at a Benjamini–Hochberg-adjusted *p* value of <0.05. For cell counting and the experimenter was blind to the condition or genotype. For all images displayed, contrast and brightness are applied in a linear manner across the entire image. For immunohistochemistry, micrographs are representative of ≥3 slices. For all graphs error bars relate to ± s.e.m., with the exception of enrichment analysis, where the 95% confidence intervals of the odds ratio are shown.

### Reporting summary

Further information on research design is available in the Nature Research Reporting Summary linked to this article.

## Supplementary information

Supplementary Information

Description of Additional Supplementary Files

Supplementary Data 1

Supplementary Data 2

Supplementary Data 3

Supplementary Data 4

Supplementary Data 5

Supplementary Data 6

Supplementary Data 7

Supplementary Data 8

Supplementary Data 9

Supplementary Data 10

Supplementary Data 11

Supplementary Data 12

Reporting summary

## Data Availability

RNA-seq data is generated in this study available from the European Nucleotide Archive (accession number: E-MTAB-10985, https://www.ebi.ac.uk/arrayexpress/experiments/E-MTAB-10985). Source data are provided with this paper.

## Source data

Source Data

## Code availability

The Python tool to disambiguate mixed-species high-throughput sequencing reads according to species is available at https://github.com/biomedicalinformaticsgroup/Sargasso.
