# Supplementary material for: Reactive astrocytes acquire neuroprotective as well as deleterious signatures in response to Tau and Aß pathology
Source: Nat Commun. 2022 Jan 10;13:135. doi: 10.1038/s41467-021-27702-w (PMC8748982; doi:10.1038/s41467-021-27702-w)
Supplement: Supplementary file 1 — Supplementary Information [file 41467_2021_27702_MOESM1_ESM.pdf]

Supplementary Figures

Supplementary Figure 1

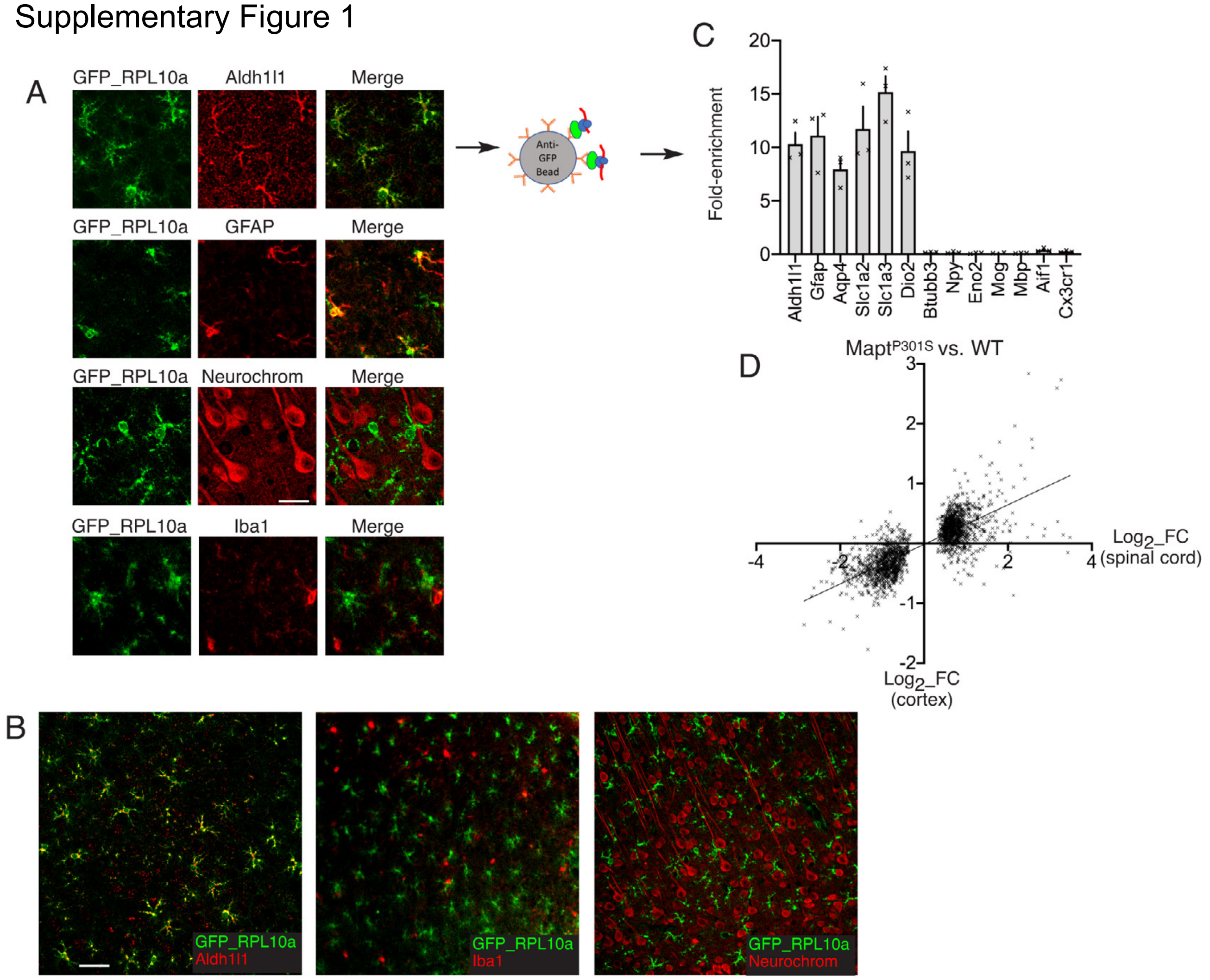

Supplementary Figure 1, related to Figure 1. A,B) Example pictures of cortical slices from Aldh1l1_eGFP-RPL10a mice processed for immunofluorescence for GFP plus either astrocyte-specific Aldh1l1 and Gfap, neuron-specific Neurochrom, or microglia-specific Iba1 markers; scale bar 20 µm (A), scale bar: 50 µm (B). **C)** Aldh1l1_eGFP-RPL10a cortices were subject to TRAP and associated RNA analysed by qPCR for expression levels of genes enriched in astrocytes (6 left-most genes), neurons (*Btubb3, Npy, Eno2*), oligodendrocytes (*Mog, Mbp*) and microglia (*Aif1, Cx3cr1*), normalised to levels in the input. Mean ± SEM shown, n=3. **D)** Log2-fold change of genes significantly changed in spinal cord MAPT^{P301S} astrocytes (measured by TRAP-seq), plotted against the corresponding log2-fold change in cortical astrocytes. Correlation coefficient r=0.77, t-=53.8 (p<1E-15), linear regression line is shown. F (1, 1950) = 2889, slope=0.33.

Supplementary Figure 2

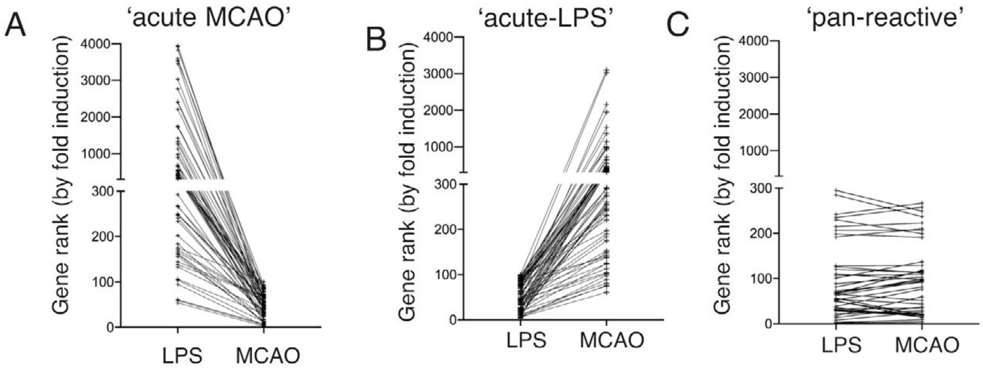

Supplementary Figure 2, related to Figure 2. A-C) Illustration of the 'acute MCAO' gene set, the 'acute-LPS' gene set and the 'pan-reactive' gene sets. We curated sets of 'acute MCAO' genes (in top 100 'acute MCAO' -induced genes [1], median rank 49; 'acute LPS' ranking >50 places lower, median rank 413, Supplementary S2A, Supplementary Data 5), sets of 'acute LPS' genes (ranked in top 100 LPS-induced genes[1], median rank 47; 'acute MCAO' ranking >50 places lower, median rank 291, Supplementary Fig. 2B, Supplementary Data 5), and pan-reactive genes (in top 250 in either type [1], <50 ranking places difference, median 'acute LPS' ranking 66, median 'acute MCAO' ranking 92, Supplementary Fig. 2C, Supplementary Data 5). The exact ranking position of each gene is shown.

Supplementary Figure 3

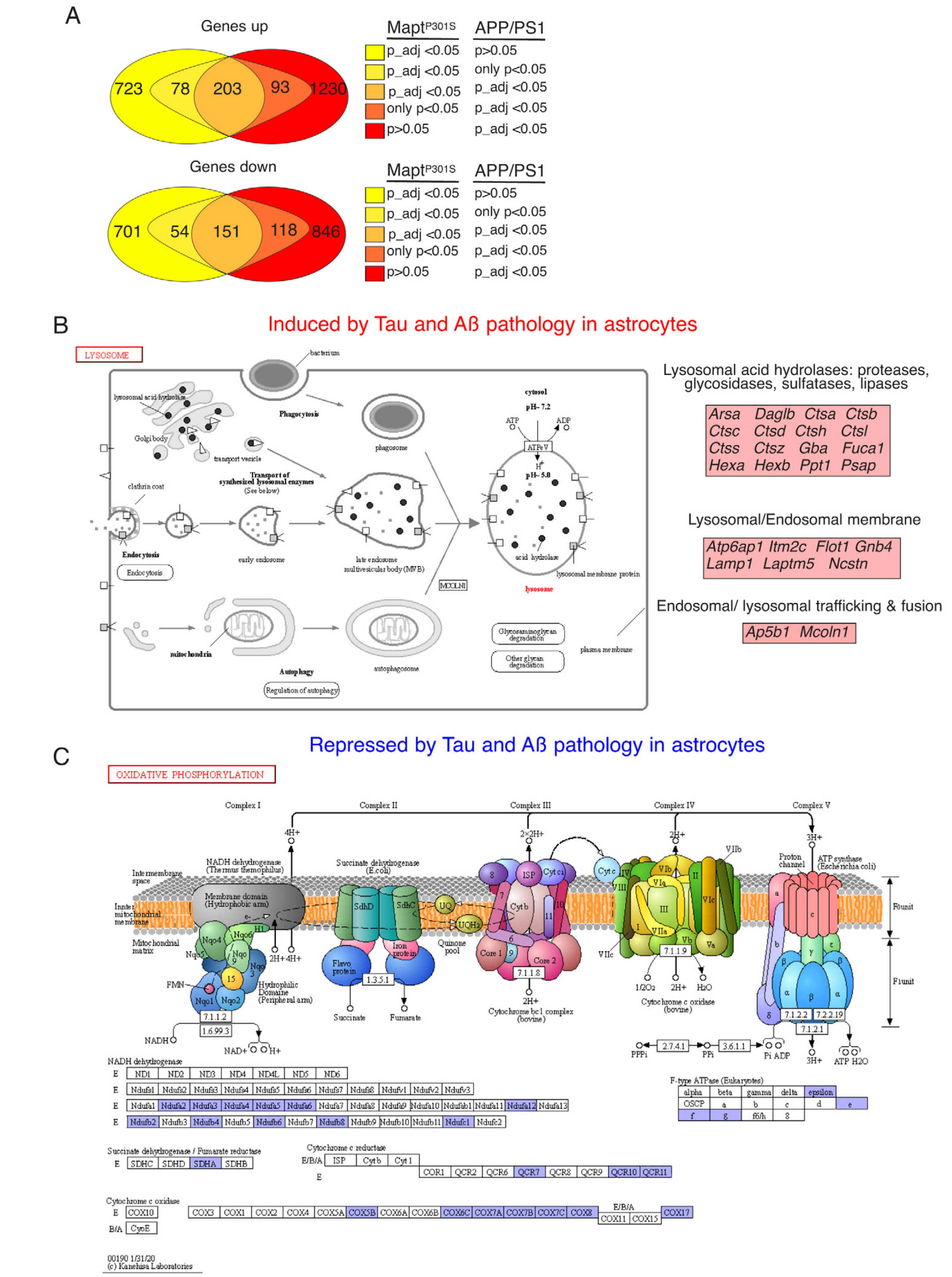

Supplementary Figure 3, related to Figure 3. A) Venn diagram of genes differentially regulated in the astrocyte translatome in models of tau and Aß pathology. See Fig. 3A also. NB. "only p<0.05" means that p<0.05 but Benjamini-Hochberg adjusted p-value >0.05. **B)** Reproduction of the KEGG lysosome pathway [2], with inclusion of the lysosomal genes induced in astrocytes in both MAPT^{P301S} and APP/PS1 mice (shaded in red). **C)** Reproduction of the KEGG oxidative phosphorylation pathway [2], with genes repressed in astrocytes in both MAPT^{P301S} and APP/PS1 mice shaded in blue.

Supplementary Figure 4

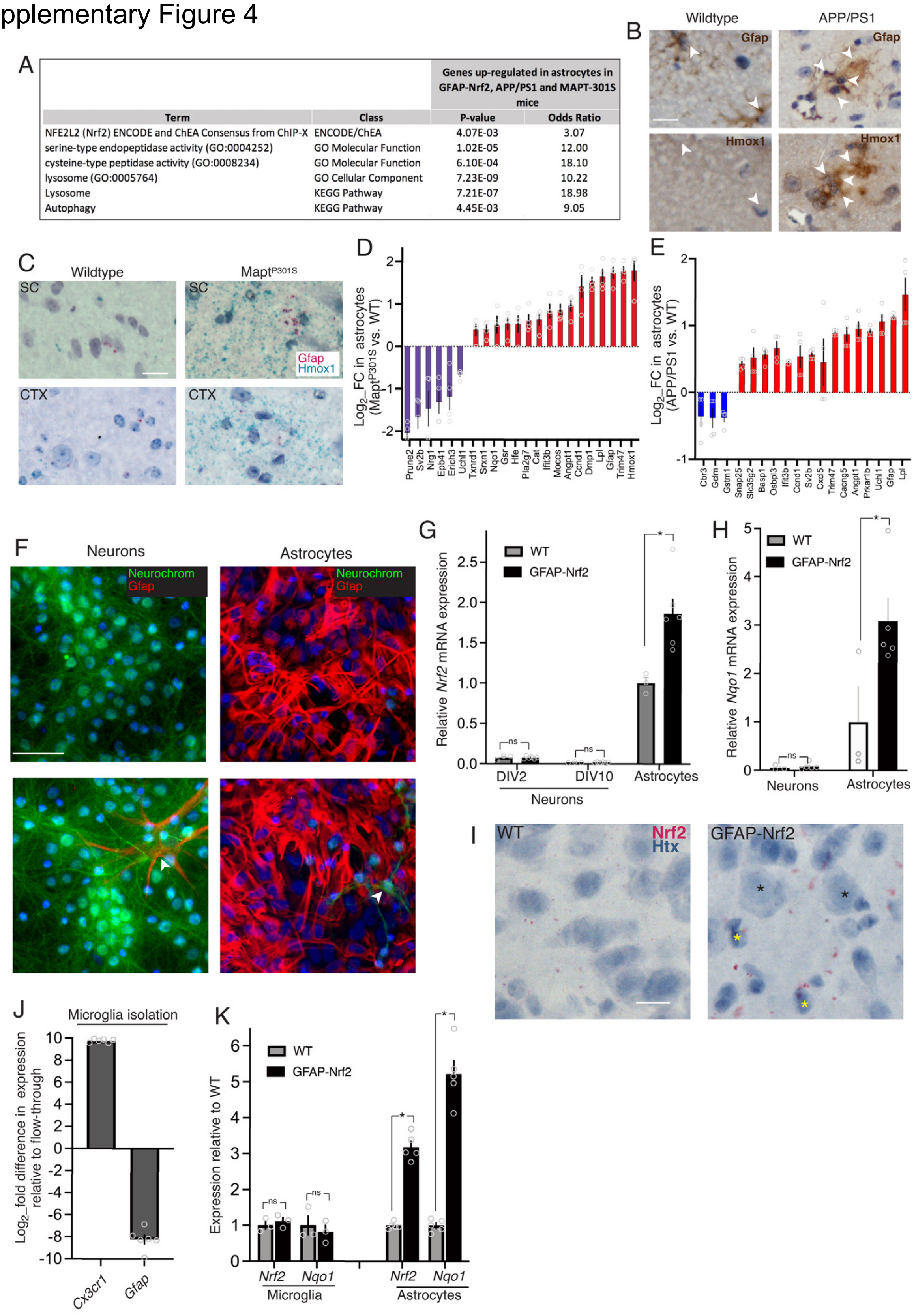

Term	Class	Genes up-regulated in astrocytes in GFAP-Nrf2, APP/PS1 and MAPT-301S mice P-value	Odds Ratio
NFE2L2 (Nrf2) ENCODE and ChEA Consensus from ChIP-X	ENCODE/ChEA	4.07E-03	3.07
serine-type endopeptidase activity (GO:0004252)	GO Molecular Function	1.02E-05	12.00
cysteine-type peptidase activity (GO:0008234)	GO Molecular Function	6.10E-04	18.10
lysosome (GO:0005764)	GO Cellular Component	7.23E-09	10.22
Lysosome	KEGG Pathway	7.21E-07	18.98
Autophagy	KEGG Pathway	4.45E-03	9.05

Supplementary Figure 4, related to Figure 4. A) For the subset of 51 Aß/Tau-induced astrocytic genes that were also induced in GFAP-Nrf2 astrocytes ($p<0.05$) enrichment analysis was performed and selected results shown. **B)** Sequential cortical slices (5 µm) were subject to immunohistochemistry for Gfap and Hmox1. Scale bar: 10 µm **C)** RNAscope analysis of *Gfap* and Hmox1 in late-stage spinal cord from MAPT^{P301S} mice vs. control. Scale bar: 10 µm **D,E)** Genes induced > 2-fold in GFAP-Nrf2 astrocytes

and significantly regulated (up or down) in the MAPT^{301S} and APP/PS1 astrocyte translatome data sets are shown, and the magnitude and direction of their regulation shown. Mean ± SEM shown here and throughout the figure. For both models this reflects a significant up-regulation of the gene sets (2-way ANOVA). Main effect of genotype: F (1, 153) = 48.67, p=8.6E-11 (D); F (1, 125) = 75.93, p=1.5E-14 (E), n=4 mice per genotype. **F)** Neuron and astrocyte enriched cultures prepared as described [3] and subject to immunofluorescent staining for Gfap and Neurochrom. Upper panels show typical fields of view. Lower panels show fields of view with rare contaminating cells as evidence of the efficacy of the other antibody staining. Scale bar: 50 µm. **G,H)** RNA extracted from neuron and astrocyte cultures described in (F) were analysed by qPCR for Nrf2 mRNA (G) and Nqo1 (H). The neurons analysed in (H) were at DIV10. G: F (1, 21) = 10.10, p=0.0045 (2-way ANOVA), *p=5.3E-05 (Bonferroni's post-hoc test, n=3). (H): F (1, 12) = 6.421, p=0.026 (2-way ANOVA), *p=0.0083 (Bonferroni's post-hoc test, n=5). **I)** Example images of RNAscope analysis of Nrf2 mRNA levels in WT and GFAP-Nrf2 slices, counterstained with hematoxylin (HTX). Black asterisks denote neurons, yellow denote glia. Scale bar: 10 µm. **J)** Validation of microglia sorted by MACS (anti-Cd11b antibody) and RNA extracted from these cells and those from the flow-through and expression of Cx3cr1 and Gfap measured and the microglial expression expressed as a fold-enrichment from levels in the flow-through (n=6: 3 WT and 3 GFAP-Nrf2 mice, both genotypes showed the same level of enrichment). **K)** For microglia sorted in (J) levels of Nrf2 and Nqo1 were assessed and expressed relative to levels in the microglia from WT mice ('ns' p-values: 0.48, 0.60, unpaired 2-tailed t-test, n=3 per genotype). For comparison, levels of Nrf2 and Nqo1 in astrocytes sorted from WT and GFAP-Nrf2 mice are shown.*p=9.0E-05, 0.0002 (unpaired 2-tailed t-test, n=5 per genotype).

Supplementary Figure 5

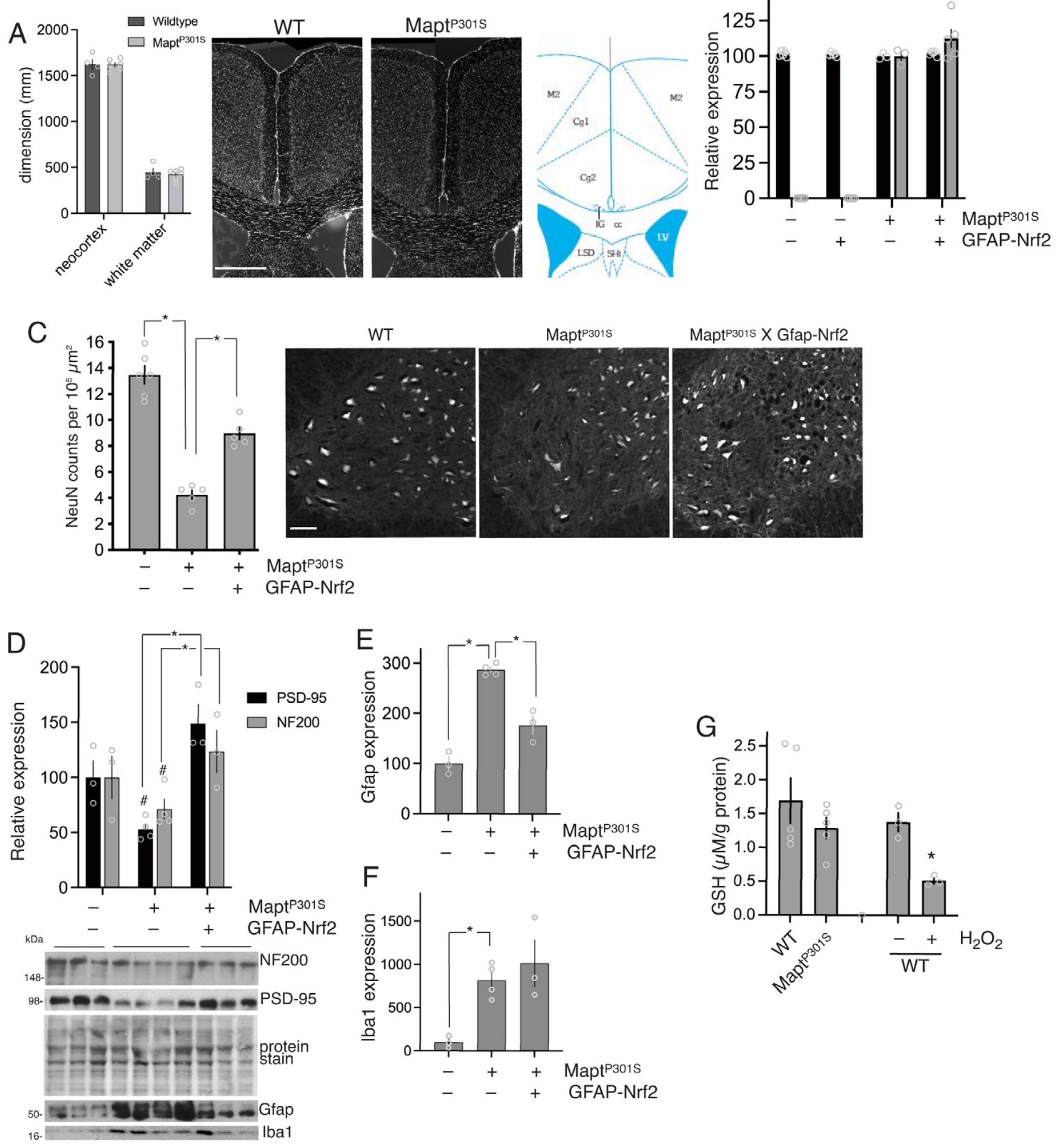

Supplementary Figure 5, related to Figure 5. A) Coronal sections (n=4 (WT; n=5 ($MAPT^{P301S}$). Mean ± SEM here and throughout. Central panels: e.g. images were stained with bisbenzimide (Scale bar: 500 µm). Right: representation of approximate coordinates: Bregma 0.62mm, interaural 4.42mm. **B)** Mouse *Mapt* and human *MAPT* RNA-seq reads separated using our species-specific RNA-seq read disambiguation workflow Sargasso [4] and expressed as a % of the level in the $MAPT^{P301S}$ mouse (n=4). **C)** Density of NeuN-positive cells in the ventral spinal cord (lamina 9). 3 slices were analysed per mouse to give a single data point. (n=3 (WT); n=6 ($MAPT^{P301S}$); n=5 ($MAPT^{P301S}$_X_GFAP-Nrf2). 1-way ANOVA (F(2, 13)=70.12, p=1.1E-07) followed by Bonferroni's post-hoc test, p=7.4E-08, 0.0002. Right: example picture (Scale: 150 µm). **D-F)** Cortical protein extracts were taken from mice of the indicated genotypes, analysed by western blot and normalised to protein levels (Memcode). 2-way ANOVA, (F (2, 14) = 14.71, p=0.0004) followed by Bonferroni's post-hoc test, *p=0.0004, 0.0349; # p=0.0295, 0.0349 (relative to WT) (D). 1- way ANOVA, (F (2, 7)=62.74, p=3.4E-05 followed by Bonferroni's post-hoc test, p=3.4E-05, 0.001 (E); 1- way ANOVA, (F (2, 7)=8.58, p=0.012 followed by Bonferroni's post-hoc test, p=0.041 (F); n=3 WT,4 $MAPT^{P301S}$; and 3 $MAPT^{P301S}$_X_GFAP-Nrf2. **G)** Spinal cord extracts were assayed for reduced glutathione (GSH) levels. The first two columns represent extracts taken at 5 months (n=5 per genotype). The last two columns represent a positive control whereby cortical tissue was exposed to acute oxidative stress (250 µM H_2O_2) and showed a loss of GSH levels *p=0.0038 (unpaired t-test, n=3).

Supplementary Figure 6

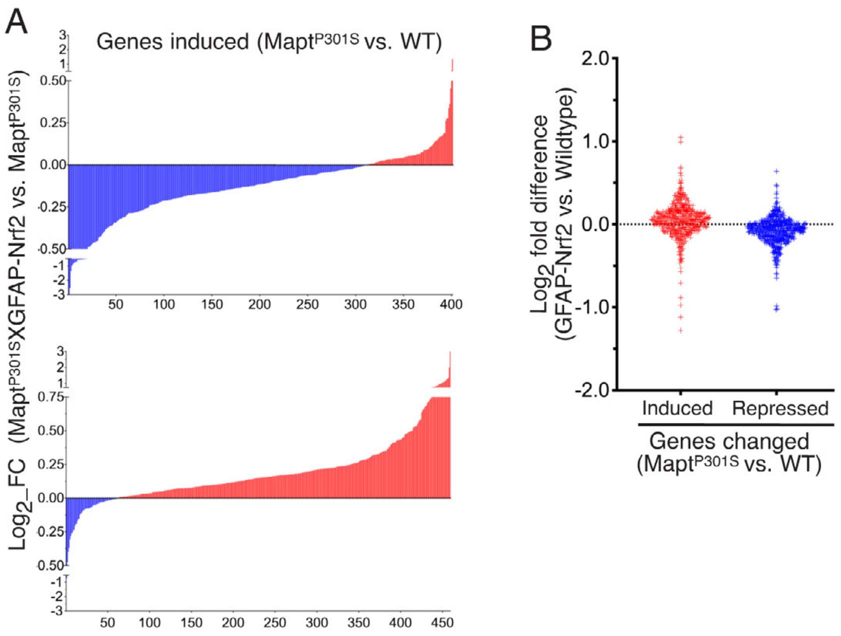

Supplementary Figure 6, related to Figure 6. A) For genes induced ($MAPT^{P301S}$ vs WT, upper) and repressed ($MAPT^{P301S}$ vs WT, lower) as highlighted in Fig. 6A, the effect of GFAP-Nrf2 is shown (i.e. $MAPT^{P301S}$_X_GFAP-Nrf2 vs. $MAPT^{P301S}$). Note how $MAPT^{P301S}$-induced genes are lowered by GFAP-Nrf2 expression, and $MAPT^{P301S}$ -repressed genes are elevated by GFAP-Nrf2 expression. A ratio paired t-test was performed on the expression (FPKM) in the $MAPT^{P301S}$ cortex vs. expression in the $MAPT^{P301S}$_X_GFAP-Nrf2 cortex: t=7.51, df=400, p=3.89E-13 (upper); t=9.97, df=458, p=2.66E-21 (lower). **B)** Difference in basal expression (GFAP-Nrf2 vs. WT) of genes induced (red) or repressed (blue) in the $MAPT^{P301S}$ mouse (vs. WT, i.e. those genes highlighted in Fig. 6A). Minimal changes are observed.

Supplementary Figure 7

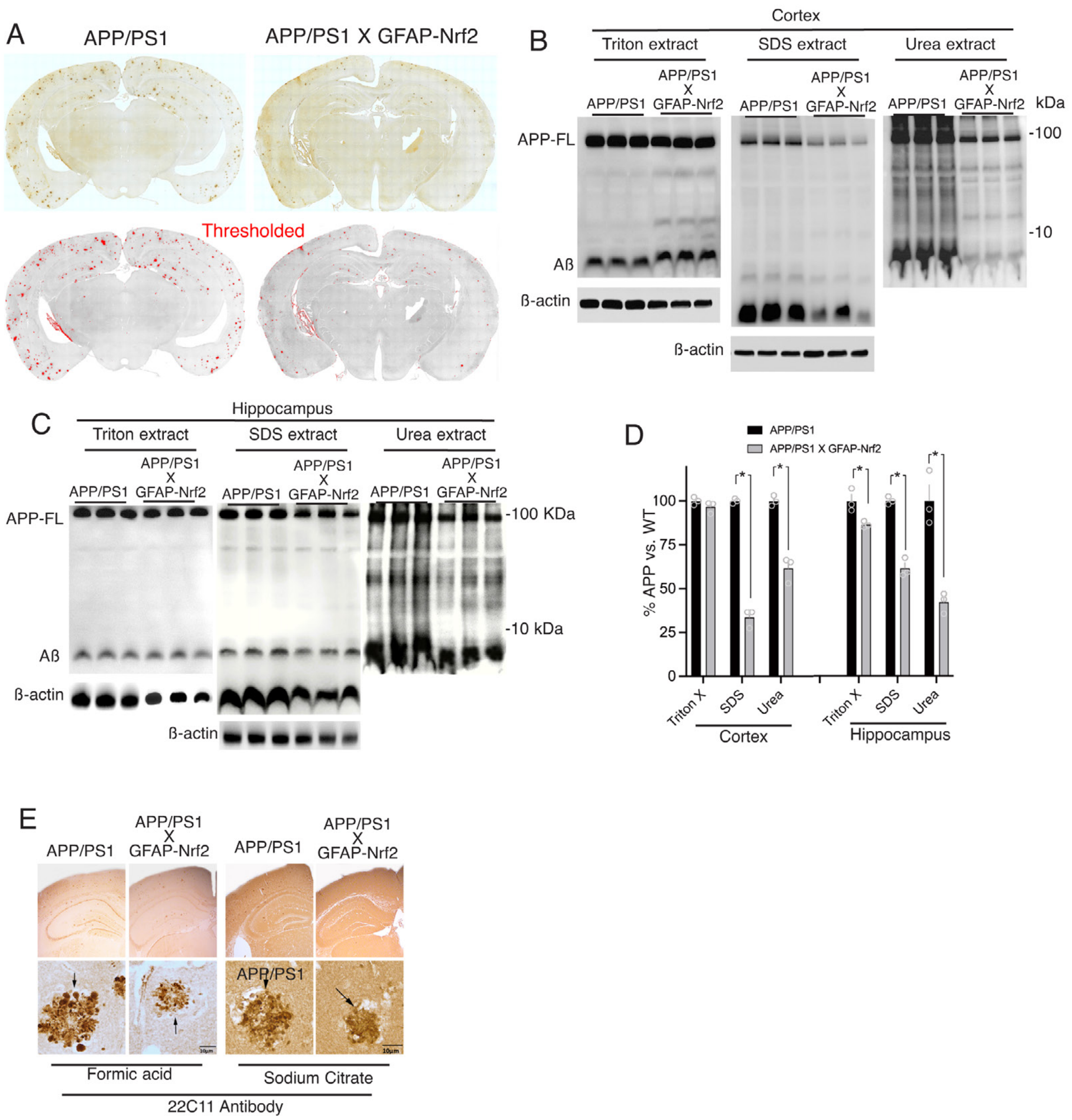

Supplementary Figure 7, related to Figure 7. A) Upper: example coronal brain sections stained for plaques using the 6E10 antibody as in Fig. 7B. Lower: example of the thresholding of staining intensity used to calculate plaque number, size and overall plaque area (Fig. 7E,F). **B-D)** Tissue fractionations from cortex and hippocampus were prepared by sequential ultracentrifugation using Triton^{TM} X-100-lysis buffer (Triton extract), Triton^{TM} X-100-lysis buffer + SDS (SDS extract), and urea (urea extract). Fractions from the cortex (B) and hippocampus (C) were resolved using a 10% Tris-Tricine polyacrylamide gel and blotted with 6E10 antibody. Quantitation is shown in (D), expressed as a % relative to wild-type, and normalized to actin loading control (with the exception of the urea extract). Mean ± SEM shown. *p values: 3.1E-05, 0.001, 0.025, 0.0003, 0.0037 (2-tailed unpaired t-test, n=3). E) Example images of brain slices processed as per Fig. 7B but using an antibody (22C11) that detects non-Aß, unprocessed AP from neuritic dystrophies caught within plaques. Scale bar: 10 µm.

Supplementary Figure 8

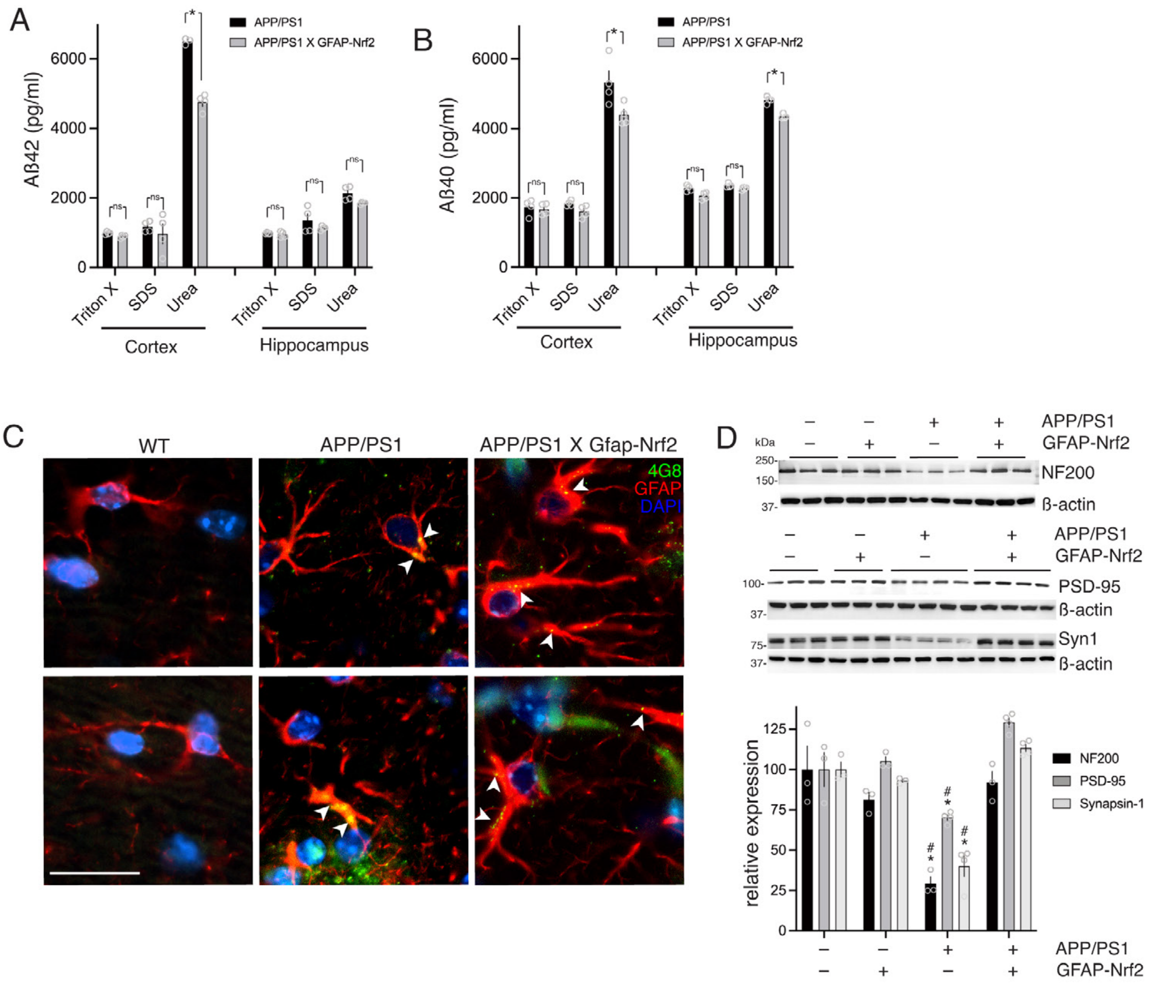

Supplementary Figure 8, related to Figure 8. A,B) The level of human Aβ$_{1-42}$ (A) and Aβ$_{1-40}$ (B) in Triton-X, SDS, and urea fractions in cortex and hippocampus at 4 months was quantified by sandwich ELISA. Mean ± SEM shown here and throughout the figure. 2-ANOVA for Aβ42 (genotype effect): F (1, 35) = 42.6, p=3.8E-11; 'ns' values: >0.99, >0.99, >0.99, 0.97, 0.48 (Bonferroni's post-hoc test). 2-ANOVA for Aβ40 (genotype effect in S8B): F (1, 36) = 22.9, p=2.9E-05. *p=2.5E-05, 0.049; 'ns' values: >0.99 for all (Bonferroni's post-hoc test). N=4 per genotype. **C)** Immunofluorescent double-labelling using antibodies against GFAP-positive astrocytes and the Aß40/22 (using the 4G8 antibody). Arrows indicate colocalization of 4G8-positive puncta and GFAP immunoreactivity. Scale bar: 20 µm**. D)** Cortical protein extracts were taken from mice of the indicated genotypes and analysed by western blot for the indicated proteins. [NF200, n=3 (all genotypes); PSD95 and Syn1, n=3 (WT, GFAP-Nrf2), n=4 (APP/PS1, APP/PS1_X_GFAP-Nrf2)]. NF200: 2-way ANOVA (F (1, 8) = 12.1, p=0.008 (APP/PS1 effect); F (1, 8) = 6.53, p=0.034 (GFAP-Nrf2 effect); F (1, 8) = 22.33, p=0.0015 (genotype interaction). PSD-95: 2-way ANOVA (F (1, 10) = 0.34, p=0.57 (APP/PS1 effect); F (1, 10) = 41.14, p=7.7E-05 (GFAP-Nrf2 effect); F (1, 10) = 28.83, p=0.0003 (genotype interaction). Synapsin-1: 2-way ANOVA (F (1, 10) = 19.9, p=0.0012 (APP/PS1 effect); F (1, 10) = 41.14, p=2.1E-05 (GFAP-Nrf2 effect); F (1, 10) = 81.8, p=4.0E-06 (genotype interaction). *p=0.0008, 0.0036, 4.8E-06 (APP/PS1 vs WT), $^{\#}$p=0.0018, 8.3E-06, 3.7E-07 (APP/PS1 vs. APP/PS1_X_GFAP-Nrf2).

Supplementary Figure 9

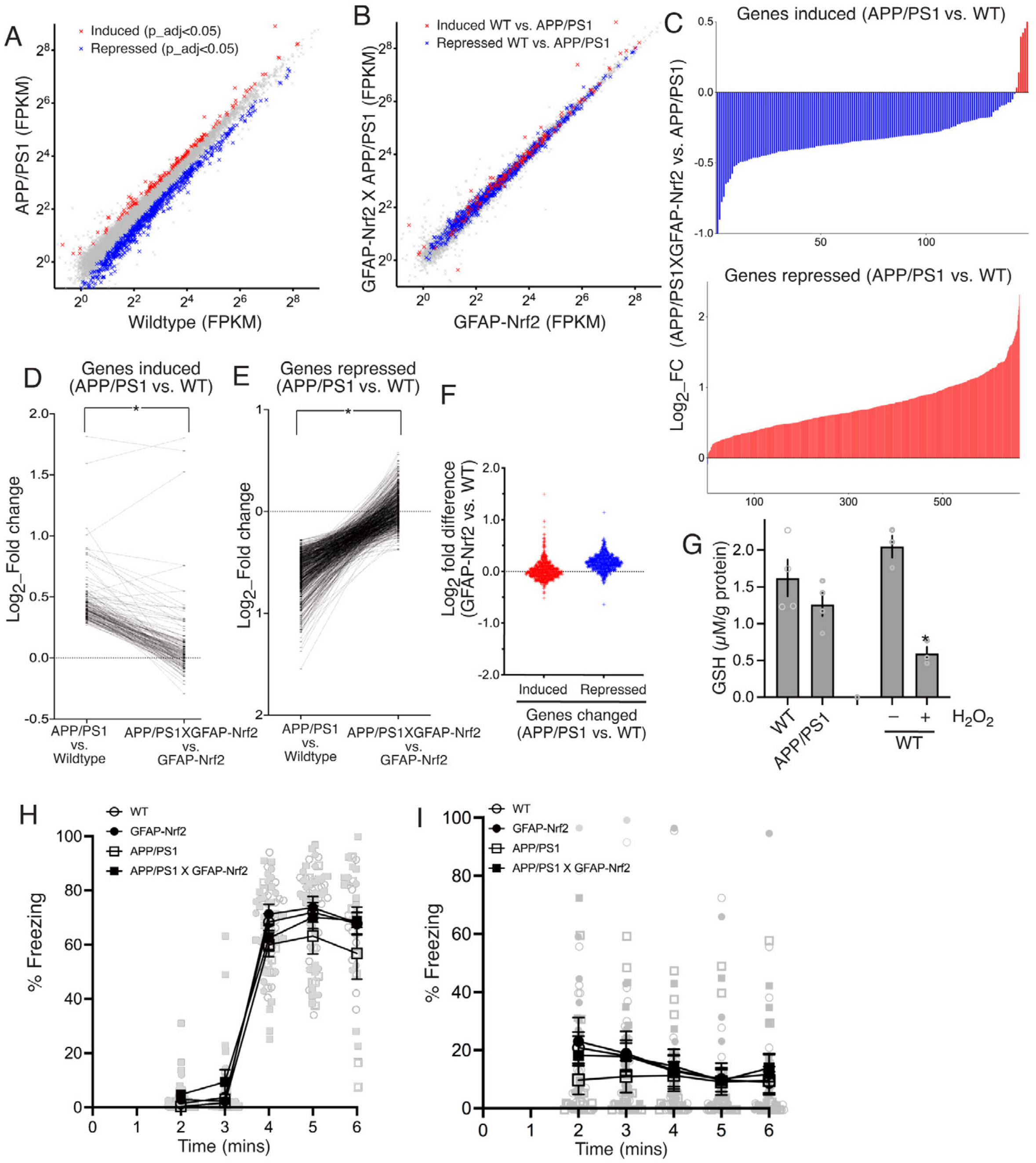

Supplementary Figure 9, related to Figure 9. A) RNA-seq analysis of the cortex (APP/PS1 vs WT). Genes induced (red) and repressed (blue) are highlighted (Benjamini-Hochberg adjusted p-value<0.05, n=40. **B)** RNA-seq analysis of early-stage neocortex (APP/PS1_X_GFAP-Nrf2 vs. GFAP-Nrf2). Genes induced (red) and repressed (blue) from Supplementary Fig. 9A are highlighted; n=4. **C)** For genes induced (APP/PS1 vs WT, upper) and repressed (APP/PS1 vs WT, lower) as highlighted in Supplementary Fig. 9A, the effect of GFAP-Nrf2 is shown (i.e. APP/PS1_X_GFAP-Nrf2 vs. APP/PS1). Note how Aß-induced genes are lowered by GFAP-Nrf2 expression, and Aß-repressed genes are elevated by GFAP-Nrf2 expression. A ratio paired t-test was performed on the expression (FPKM) in the APP/PS1 hippocampus vs. expression in the APP/PS1_X_GFAP-Nrf2 cortex: t=1.90, df=148, p=0.059 (upper); t=73.35, df=662, *p<1E-15 (lower), n=4. **D,E)** A comparison of the fold-change in gene expression caused by APP/PS1 expression in an otherwise WT background (i.e. APP/PS1 vs. WT) compared to the fold-change in gene expression caused by APP/PS1 expression against a GFAP-Nrf2 background (i.e. APP/PS1 vs. APP/PS1_X_GFAP-Nrf2). Genes induced (D, 147 genes) or repressed (E, 663 genes) (i.e. genes

highlighted in Supplementary Fig. 9A) are shown. Note how GFAP-Nrf2 represses the induction of Aß-induced genes and inhibits the repression of Aß-repressed genes. T=7.230, df=146, *p=2.5E-11 (D); t=54.6, df=662, *p<1E-15 (E). **F)** Difference in basal expression (GFAP-Nrf2 vs. WT) of genes induced (red) or repressed (blue) in the APP/PS1 mouse (vs. WT, i.e. those genes highlighted in Supplementary Fig. 9A). Minimal changes are observed. **G)** Cortical extracts of the indicated genotypes were assayed for reduced glutathione (GSH) levels. The first two columns represent extracts taken at 12 months (n=4 per genotype) and show no difference. The last pair of columns represent a control whereby cortical tissue was exposed to oxidative stress (250 µM H_2O_2) and showed a loss of GSH levels. Mean ± SEM show; *p=0.0038 (unpaired t-test, n=3). **H)** Fear conditioning test at 7 months. Data are presented as percent freezing during cue testing in 1 min bins starting 60 s after being placed in the chamber (mean ± SEM): WT(n=19); GFAP-Nrf2 (n=16); APP/PS1 (n=9); APP/PS1_X_GFAP-Nrf2 (n=21) mice. Average % freezing across bins 4-6 was calculated for a one-way ANOVA (main genotype effect): (F (3, 61) = 1.01, p = 0.3943). **I)** Context training at 9 months. Data are presented as percent freezing during context testing in 1 min bins starting 60 s after being placed in the chamber (mean ± SEM): WT (n=18); GFAP-Nrf2 (n=13); APP/PS1 (n=13); APP/PS1_X_GFAP-Nrf2 (n=11) mice. Average % freezing across all bins calculated for a one-way ANOVA (main genotype effect): (F (3, 61) = 0.20 , p = 0.89).

Supplementary References

1 Zamanian, J. L. *et al.* Genomic analysis of reactive astrogliosis. *J Neurosci* **32**, 6391-6410, doi:32/18/6391 [pii]
10.1523/JNEUROSCI.6221-11.2012 (2012).

2 Kanehisa, M. & Goto, S. KEGG: kyoto encyclopedia of genes and genomes. *Nucleic Acids Res* **28**, 27-30, doi:10.1093/nar/28.1.27 (2000).

3 Bell, K. F. *et al.* Neuronal development is promoted by weakened intrinsic antioxidant defences due to epigenetic repression of Nrf2. *Nat Commun* **6**, 7066, doi:ncomms8066 [pii]
10.1038/ncomms8066 (2015).

4 Qiu, J. *et al.* Mixed-species RNA-seq for elucidating non-cell-autonomous control of gene transcription. *Nat Protoc* **13**, 2176-2199, doi:10.1038/s41596-018-0029-2 (2018).
