# Supplementary material for: Reactive astrocytes acquire neuroprotective as well as deleterious signatures in response to Tau and Aß pathology
Source: Nat Commun. 2022 Jan 10;13:135. doi: 10.1038/s41467-021-27702-w (PMC8748982; doi:10.1038/s41467-021-27702-w)
Supplement: Supplementary file 2 — Description of Additional Supplementary Files [file 41467_2021_27702_MOESM2_ESM.pdf]

Description of Additional Supplementary Files

Supplementary Data 1. Data relating to Fig. 1A-E: Astrocyte TRAP-seq data relating to WT vs. Mapt^{P301S} mice at early stage disease.

Supplementary Data 2. Data relating to Fig. 1A-E: Astrocyte TRAP-seq data relating to WT vs. Mapt^{P301S} mice at late stage disease.

Supplementary Data 3. Data relating to Fig. 1F-J: Astrocyte TRAP-seq data relating to WT vs. APP/PS1 mice at early stage disease.

Supplementary Data 4. Data relating to Fig. 1F-J: Astrocyte TRAP-seq data relating to WT vs. APP/PS1 mice at late stage disease.

Supplementary Data 5. Data relating to Supplementary Fig. 2A-C: Illustration of the ‘acute MCAO’ gene set, the ‘acute-LPS’ gene set and the ‘pan-reactive’ gene sets.

Supplementary Data 6. Data relating to Fig, 3A and Supplementary Fig. 3: Set of genes significantly upregulated in both Mapt^{P301S} and APP/PS1 models, and set of genes down-regulated in both Mapt^{P301S} and APP/PS1 models.

Supplementary Data 7. Data relating to Fig. 4B. RNA-seq analysis of astrocytes sorted from GFAP-Nrf2 mice, vs. WT.

Supplementary Data 8. Data relating to Fig. 6A,B: RNA-seq analysis of the neocortex (WT, Mapt^{P301S}, GFAP-Nrf2, Mapt^{P301S}_X_GFAP-Nrf2). Genes induced and repressed (Mapt^{P301S} vs. WT) are shown.

Supplementary Data 9. Data relating to Fig. 9A,B: RNA-seq analysis of hippocampus (WT, APP/PS1, GFAP-Nrf2, APP/PS1_X_GFAP-Nrf2). Genes induced and repressed (APP/PS1 vs. WT) are shown.

Supplementary Data 10. Data relating to Supplementary Fig. 9A,B: RNA-seq analysis of cortex (WT, APP/PS1, GFAP-Nrf2, APP/PS1_X_GFAP-Nrf2). Genes induced and repressed (APP/PS1 vs. WT) are shown.

Supplementary Data 11. Data relating to Fig. 9G: Genes down-regulated by Aß pathology (APP/PS1 vs. WT, p_adj<0.05, Fig. 9A) that were also present in analysis of genes up-regulated by astrocytic Nrf2 in the APP/PS1 mouse (APP/PS1_X_GFAP-Nrf2 vs. APP/PS1, Fig. 9G).

Supplementary Data 12. Data relating to Fig. 9H. Aß pathology induces tissue-wide deficits in four primary areas that are rescued by astrocytic Nrf2.
