# Supplementary material for: Reactive astrocytes acquire neuroprotective as well as deleterious signatures in response to Tau and Aß pathology
Source: Nat Commun. 2022 Jan 10;13:135. doi: 10.1038/s41467-021-27702-w (PMC8748982; doi:10.1038/s41467-021-27702-w)
Supplement: Supplementary file 15 — Reporting summary [file 41467_2021_27702_MOESM15_ESM.pdf]

nature portfolio

Corresponding author(s): Giles Hardingham, Siddharthan Chandran, Jeffrey Johnson

Last updated by author(s): Nov 16, 2021

Reporting Summary

Nature Portfolio wishes to improve the reproducibility of the work that we publish. This form provides structure for consistency and transparency in reporting. For further information on Nature Portfolio policies, see our Editorial Policies and the Editorial Policy Checklist.

Statistics

For all statistical analyses, confirm that the following items are present in the figure legend, table legend, main text, or Methods section.

n/a	Confirmed	
☐	☒	The exact sample size (*n*) for each experimental group/condition, given as a discrete number and unit of measurement
☐	☒	A statement on whether measurements were taken from distinct samples or whether the same sample was measured repeatedly
☐	☒	The statistical test(s) used AND whether they are one- or two-sided<br>*Only common tests should be described solely by name; describe more complex techniques in the Methods section.*
☒	☐	A description of all covariates tested
☐	☒	A description of any assumptions or corrections, such as tests of normality and adjustment for multiple comparisons
☐	☒	A full description of the statistical parameters including central tendency (e.g. means) or other basic estimates (e.g. regression coefficient) AND variation (e.g. standard deviation) or associated estimates of uncertainty (e.g. confidence intervals)
☐	☒	For null hypothesis testing, the test statistic (e.g. *F*, *t*, *r*) with confidence intervals, effect sizes, degrees of freedom and *P* value noted<br>*Give P values as exact values whenever suitable.*
☒	☐	For Bayesian analysis, information on the choice of priors and Markov chain Monte Carlo settings
☒	☐	For hierarchical and complex designs, identification of the appropriate level for tests and full reporting of outcomes
☒	☐	Estimates of effect sizes (e.g. Cohen's *d*, Pearson's *r*), indicating how they were calculated

Our web collection on statistics for biologists contains articles on many of the points above.

Software and code

Policy information about availability of computer code

Data collection	Illumina Novaseq 6000 platform; Leica LAS X; ImageJ v1.53
Data analysis	Mixed-species sorting was carried out using the SARGASSO (Sargasso Assigns Reads to Genomes According to Species-Specific Origin) python tool as described (Qiu et al., 2018) (source code available: http://statbio.github.io/Sargasso). RNA-seq reads were mapped to genome sequences using STAR v2.7.0f (Spliced Transcripts Alignment to a Reference, (Dobin et al., 2013)). Per-gene read counts were summarised using featureCounts v1.6.3 (Liao et al., 2014), and differential expression analysis performed using DESeq2 v1.24.0 (Love et al., 2014).<br><br>Dobin, A., Davis, C.A., Schlesinger, F., Drenkow, J., Zaleski, C., Jha, S., Batut, P., Chaisson, M., and Gingeras, T.R. (2013). STAR: ultrafast universal RNA-seq aligner. Bioinformatics 29, 15-21.<br><br>Liao, Y., Smyth, G.K., and Shi, W. (2014). featureCounts: an efficient general purpose program for assigning sequence reads to genomic features. Bioinformatics 30, 923-930.<br><br>Love, M.I., Huber, W., and Anders, S. (2014). Moderated estimation of fold change and dispersion for RNA-seq data with DESeq2. Genome Biol 15, 550.<br><br>Qiu, J., Dando, O., Baxter, P., Hasel, P., Heron, S., Simpson, I.T., and Hardingham, G.E. (2018). Mixed-species RNA-seq for elucidating non-cellautonomous control of gene transcription. Nat Protoc 13, 2176-2199.

For manuscripts utilizing custom algorithms or software that are central to the research but not yet described in published literature, software must be made available to editors and reviewers. We strongly encourage code deposition in a community repository (e.g. GitHub). See the Nature Portfolio guidelines for submitting code & software for further information.

Data

Policy information about availability of data

All manuscripts must include a data availability statement. This statement should provide the following information, where applicable:

- Accession codes, unique identifiers, or web links for publicly available datasets
- A description of any restrictions on data availability
- For clinical datasets or third party data, please ensure that the statement adheres to our policy

RNA-seq data is generated in this study available from the European Nucleotide Archive (accession number: E-MTAB-10985, https://www.ebi.ac.uk/arrayexpress/experiments/E-MTAB-10985/). Source Data is provided in Source_Data.xls.

Field-specific reporting

Please select the one below that is the best fit for your research. If you are not sure, read the appropriate sections before making your selection.

☒ Life sciences ☐ Behavioural & social sciences ☐ Ecological, evolutionary & environmental sciences

For a reference copy of the document with all sections, see nature.com/documents/nr-reporting-summary-flat.pdf

Life sciences study design

All studies must disclose on these points even when the disclosure is negative.

Sample size	Sample size was estimated utilising 3R (Reduction, Replacement and Refinement) principles, with sample size numbers calculated to achieve satisfactory power based on the variance of degeneration reported previously in the MAPT-P301S tauopathy mouse, and cognitive deterioration in the APP/PS1 mouse, as described in the following references:<br><br>D. W. Hampton,et al. Journal of Neuroscience 2010, 30, 9973.<br>B. Allen, et al. J. Neurosci. 2002, 22, 9340.<br>R. Radde, et al. EMBO Rep. 2006, 7, 940.
Data exclusions	No samples or animals were excluded from the analysis.
Replication	All findings were replicated. Replication numbers are indicated within the figure legends. All replicates used for statistical purposes are biological replicates and not technical replicates, and all replications were included within the presented data.
Randomization	Since group allocation was based on genotype randomization was not needed or appropriate.
Blinding	Blinding was done where appropriate, with per-experiment statement made in Materials and Methods. In particular, transcriptomic analysis and associated workflows; imaging studies; molecular assays; and animal functional and behavioral studies were blinded until the point of data-analysis. The fear conditioning was blinded. Cages had mixed genotypes and mice were microchipped. The person testing the mice in the Behavioral Core scanned the mice prior to testing, and noted the microchip number without having access to the genotype.

Reporting for specific materials, systems and methods

We require information from authors about some types of materials, experimental systems and methods used in many studies. Here, indicate whether each material, system or method listed is relevant to your study. If you are not sure if a list item applies to your research, read the appropriate section before selecting a response.

Materials & experimental systems

n/a	Involved in the study	
☐	☒	Antibodies
☒	☐	Eukaryotic cell lines
☒	☐	Palaeontology and archaeology
☐	☒	Animals and other organisms
☒	☐	Human research participants
☒	☐	Clinical data
☒	☐	Dual use research of concern

Methods

n/a	Involved in the study	
☒	☐	ChIP-seq
☒	☐	Flow cytometry
☒	☐	MRI-based neuroimaging

Antibodies

Antibodies used	A full list of antibodies is found in the materials and methods and repeated here:

β-Amyloid 1-16, (6E10) 1:1,000 (WB, IHC, IF)Biolegend 803003
β-Amyloid 17-24 (4G8) 1:500 (IF) Biolegend 800708
APP A4 66-81, (22C11) 1:1,000 (WB, IHC) Millipore MAB-348
APP 676-695 (C1/6.1) 1:10,000 (WB) Biolegend 802801
BACE1 1:1,000 (WB) GeneScript A00879
GFAP (GA5) 1:10,000 (WB) 1:1000 (IF) Sigma G 3892
GFAP GA5-Cy3 1:500 (IF) Sigma MAB3402
IBA1 1:1,000 (WB), I:500 (IF) Wako 019-19741
NQO1 1:1,000 WB Abcam ab34173
NQO1 (A180) 1:200 (IF) Santa Cruz sc-32793
GCLM 1:1,000 (WB) Dr. Terry Kavanagh University of Washington
β-Actin (8H10D10) 1:10,000 (WB) Cell Signaling Technology 3700
NeuN 1:400(IF) Millipore Bioscience MAB377
Phosphotau AT8 1:1000 (IF) Autogen Bioclear 90206
Neurochrom-Cy3 1:500 (IF) Millipore Bioscience ABN2300C3
Anti-Aldh1l1 1:500 (IF) Abcam ab87117
SQSTM1/p62 1:1,000 (WB) Abnova H00008878-B01
NF200 (NE14) 1:500 (WB) EMD Millipore N5389
NF200 (N52) 1:1000 (WB) EMD Millipore MAB5266
PSD95 1:1000)WB) Abcam ab12093
Synapsin1 1:1000 (WB) Synaptic Systems 106 103

Validation

Full validation information can be found at the manufacturer's websites as provided above. Antibodies were used in the concentration range recommended by the manufacturer following final validation in the lab to confirm concentration and to exclude non-specific signals using appropriate species-matched secondary antibodies. Validation summary:
β-Amyloid 1-16, (6E10) 1:1,000 (WB, IHC, IF)Biolegend 803003: western detection of of Ab1-440/42 peptide, recombinant APP and APP in brain lysate
β-Amyloid 17-24 (4G8) 1:500 (IF) Biolegend 800708: IHC detection of β-Amyloid in AD tissue, ELISA detection of recombinant Aß
APP A4 66-81, (22C11) 1:1,000 (WB, IHC) Millipore MAB-348 Recognised APP and Aß-PMID:21085580
APP 676-695 (C1/6.1) 1:10,000 (WB) Biolegend 802801:Western blot of purified anti-APP C-Terminal Fragment antibody
BACE1 1:1,000 (WB) GenScript A00879: Anti-BACE1 (46-65) Polyclonal Antibody. Detects correct MW
GFAP (GA5) 1:10,000 (WB) 1:1000 (IF) Sigma G 3892. Monoclonal Anti-GFAP has been tested for immuno- cytochemical localization of GFAP in human, pig, and rat tissues.
GFAP GA5-Cy3 1:500 (IF) Sigma MAB3402. As above
IBA1 1:1,000 (WB), I:500 (IF) Wako 019-19741. Immunogen: synthetic peptide C-terminal of Iba1. Used in many papers-see https://labchem-wako.fujifilm.com/us/product_data/docs/00055446_doc03.pdf
NQO1 1:1,000 WB Abcam ab34173
NQO1 (A180) 1:200 (IF) Santa Cruz sc-32793. Antibody citations: https://www.scbt.com/p/nqo1-antibody-a180. Detects NQO1 in Western blot analysis of NQO1 expression in Hep G2 (A), A549 (B), AN3 CA (C) and SK-MEL-24 (D) whole cell lysates.
GCLM 1:1,000 (WB) Dr. Terry Kavanagh University of Washington
β-Actin (8H10D10) 1:10,000 (WB) Cell Signaling Technology 3700. Identifies single 42kDa band in multiple cell lysates.
NeuN 1:400(IF) Millipore Bioscience MAB377. Monoclonal antibody to vertebrate neuron-specific nuclear protein called NeuN Developmentally, immunoreactivity is first observed shortly after neurons have become postmitotic, no staining has been observed in proliferative zones. The immunohistochemical staining is primarily localized in the nucleus of the neurons with lighter staining in the cytoplasm
Phosphotau AT8 1:1000 (IF) Autogen Bioclear 90206. Validated in ± okadaic acid treated tau-expressing cells.
Neurochrom-Cy3 1:500 (IF) Millipore Bioscience ABN2300C3. Mixture of `ntibodies specific to axons dendrites, nucleus, and the cell body of neurons. References 1. Long, K. et al. (2008) Abstract; Society for Neuroscience Annual Meeting. 2. Speckmann, W. et al. (2011) Abstract; Society for Neuroscience Annual Meeting
Anti-Aldh1l1 1:500 (IF) Abcam ab87117. See https://www.abcam.com/ALDH1L1-antibody-Astrocyte-Marker-ab87117.html for multiple references
SQSTM1/p62 1:1,000 (WB) Abnova H00008878-B01. Antibody reactive against recombinant protein.
NF200 (NE14) 1:500 (WB) EMD Millipore N5389. Validated in brain sections localization as expected, size as expected by WB.
NF200 (N52) 1:1000 (WB) EMD Millipore MAB5266. Multiple references aat https://www.merckmillipore.com/GB/en/product/Anti-Neurofilament-200-kDa-Antibody-clone-N52,MM_NF-MAB5266
PSD95 1:1000)WB) Abcam ab12093. Detects correct size band in brain lysate
Synapsin1 1:1000 (WB) Synaptic Systems 106 103. Detects correct size band in brain synaptosome extract, and synaptic localisation by IHC.

Animals and other organisms

Policy information about studies involving animals; ARRIVE guidelines recommended for reporting animal research

Laboratory animals

Mice were group-housed in environmentally-enriched cages within standard humidity and temperature controlled rooms, with a 12-hour light dark cycle with free access to food and water. Ages of all mice used in experiments was between 3-12 months as appropriate for each relevant disease model and as stated in the text and figure legends. Strains used for experiments were APP/

	PS1de9l; MAPT-P301S, GFAP-Nrf2; Aldh1l1-EGFP-Rpl10a. All strains were on a C57B/6J background and C57B/6J mice were used for controls where appropriate. Experiments involving APP/PS1de9 mice used mixed sex. Experiments involving MAPT-P301S mice used female mice as they demonstrate less fighting and exhibit more rapid-onset pathology than males.
Wild animals	No wild animals were used in this study.
Field-collected samples	No field collected samples were used in this study.
Ethics oversight	All procedures described were performed either in the University of Edinburgh in compliance with the UK Animals (Scientific Procedures) Act 198b and University of Edinburgh regulations, and carried out under project license numbers 70/9008 and P1351480E, or in the University of Wisconsin-Madison, approved by their Institutional Animal Care and Use Committee.

Note that full information on the approval of the study protocol must also be provided in the manuscript.
